# Second-Order Systematicity of Associative Learning: A Paradox for Classical Compositionality and a Coalgebraic Resolution

**DOI:** 10.1371/journal.pone.0160619

**Published:** 2016-08-09

**Authors:** Steven Phillips, William H. Wilson

**Affiliations:** 1 Human Informatics Research Institute, National Institute of Advanced Industrial Science and Technology (AIST), Tsukuba, Ibaraki, Japan; 2 School of Computer Science and Engineering, The University of New South Wales, Sydney, New South Wales, Australia; Plymouth University, UNITED KINGDOM

## Abstract

Systematicity is a property of cognitive architecture whereby having certain cognitive capacities implies having certain other “structurally related” cognitive capacities. The predominant classical explanation for systematicity appeals to a notion of common syntactic/symbolic structure among the systematically related capacities. Although learning is a (second-order) cognitive capacity of central interest to cognitive science, a systematic ability to learn certain cognitive capacities, i.e., second-order systematicity, has been given almost no attention in the literature. In this paper, we introduce learned associations as an instance of second-order systematicity that poses a paradox for classical theory, because this form of systematicity involves the kinds of associative constructions that were explicitly rejected by the classical explanation. Our category theoretic explanation of systematicity resolves this problem, because both first and second-order forms of systematicity are derived from the same categorical construction: universal morphisms, which generalize the notion of compositionality of constituent representations to (categorical) compositionality of constituent processes. We derive a model of systematic associative learning based on (co)recursion, which is an instance of a universal construction. These results provide further support for a category theory foundation for cognitive architecture.

## Introduction

In the Spring of 2011, at the seaside town of San Jose, Spain, cognitive scientists gathered for a workshop to reassess the systematicity problem that Fodor and Pylyshyn [1] posed to connectionists more than two decades earlier. That meeting was the catalyst for a collection of articles [2] providing a diverse range of views on systematicity and its import for a theory of cognitive architecture. Though significant progress has been made on clarifying the problem [3, 4]—i.e., why systematicity necessarily follows from theoretical principles without relying on arbitrary additional assumptions to bridge explanatory gaps—consensus on an explanation appears to be as elusive as ever [5].

The problem of systematicity for cognitive science is to explain why certain cognitive capacities typically co-exist [1]; why, for example, does having the ability to identify square as the top object in a scene consisting of a square above a triangle implies having the ability to identify triangle as the top object in a scene consisting of a triangle above a square. More formally and generally, an instance of systematicity occurs when one has cognitive capacity *c*_1_ if and only if one has “structurally related” cognitive capacity *c*_2_ [6], i.e., systematicity is the partitioning of cognitive capacities into structurally equivalent classes of cognitive capacities. Though cognitive scientists may agree that underlying an instance of systematicity is a capacity to process common structure, such as the common relation *above* in the aforementioned example, they disagree over the proposed nature of such processes, e.g., symbolic versus subsymbolic [7, 8], and whether such proposals constitute an explanation for systematicity [3, 6].

Given that cognition is systematic to some significant extent, then assessing proposals against explanatory criteria is pivotal. A sufficiently general theory of cognition may afford cognitive models that support systematicity. Yet, if that same theory affords models that do not support systematicity, then the challenge is to explain why we only observe the corresponding systematic cases in the domain of interest. In short, we require an explanation of systematicity that does not rely on adjoining arbitrary (*ad hoc*) assumptions to meet explanatory gaps, i.e., auxiliary assumptions that are motivated only to fit the data, cannot be verified independently of verifying the theory, and are unconnected to the theory’s core principles [3].

Two proposals have explicitly claimed explanations for systematicity without recourse to ad hoc assumptions. The first proposal is a computational symbol systems approach [1], which we will refer to as *classical compositionality*. Classical compositionality is characterized by the notion of *tokening*: representations of constituent entities are tokened whenever the representation of its complex host entity is tokened. Each “position” within the complex representation is a domain for an inference process. Hence, if there exists a process for accessing, say, the first component of a pair, then that process extends to all constituent representations that can fill the first position. The other proposal is a mathematical category theory [9] approach [10], which we will refer to as *categorical compositionality*. Central to categorical compositionality is the (formal) notion of *universal construction*, where each and every instance in a collection of systematically-related cognitive capacities is obtained by a *morphism* that factors through (i.e., includes) a common, or shared component, called a *universal morphism*. Hence, having the universal morphism that constitutes one capacity implies having all capacities that share this morphism, assuming the morphisms that correspond to the other constituent capacities such as those that correspond to square and triangle.

### Second-order systematicity

Learning is a (second-order) cognitive capacity; a cognitive capacity that engenders other cognitive capacities. Hence, using the characterization of systematicity as equivalence classes of structurally related cognitive capacities [6], we have another form of systematicity, i.e. having *learning* capacity *l*_1_ to acquire cognitive ability *c*_1_ if and only if one has structurally related learning capacity *l*_2_ to acquire cognitive ability *c*_2_, which is referred to as *second-order systematicity* [3]. This characterization of second-order systematicity parallels the characterization of first-order systematicity. (For comparison, first-order systematicity is having cognitive capacity *c*_1_ if and only if one has structurally related cognitive capacity *c*_2_.) Aizawa [3], citing Chomsky [11], provides an example from language: a person has the capacity to learn one natural language (say, Chinese) if they have the capacity to learn another (say, German). A mundane instance of second-order systematicity can be found in experimental psychology, where subjects are paid to participate in multiple experiments involving different cognitive tasks, simply as a matter of logistics: typically, a subject can learn (by instruction) to complete one cognitive task if they can learn to complete another cognitive task. Such situations usually involve tasks designed with different materials and procedures to avoid biasing results (e.g., a word stem completion task and a mental rotation task).

The second example alludes to an important aspect of second-order systematicity that, we will show, impacts upon theories of cognitive architecture: the capacities that are learned (*c*_1_ and *c*_2_) need not be systematically related to each other; the structural relation need only occur at the second-order level, i.e. between the corresponding learning capacities (*l*_1_ and *l*_2_). An example that is pertinent to the classical explanation for systematicity is the learning (or memorization) of associations. For instance, if one has the capacity to learn that the *first day of the Japanese financial year is April 1st*, then one also has the capacity to learn that the *atomic number of carbon is 6*, at the syntactic or semantic level (see [12] for syntactic versus semantic systematicity), assuming that one already can represent the entities to be associated, i.e., Japanese financial year, April 1st, atomic weight (carbon) and the number 6. The intuition behind this example is that there need not be any internal structural relations between the entities of different associations, e.g., Japanese financial year and atomic weight (carbon). The internal structures of the respective concepts play no role in this instance of systematicity, in contrast with the square-triangle example where the internal structure is the same relation. Yet, there is an external structural relation in the sense that each fact can be considered as a mapping from a concept to a feature value. As we will elaborate upon shortly, this example is a legitimate instance of systematicity (at the second-order level) given that systematicity has been characterized as a structural equivalence relation over cognitive capacities [6].

We elaborate upon the notion of second-order systematicity of associative learning by first recalling characteristic features of associative learning. “Associative learning … is basically the learning that results from experiencing contingencies, or predictive relationships, between events” [13] (p. 18). A feature of associative learning common to humans and animals is that the contingencies are predictive, hence the principle that associative learning processes are engaged when an outcome is not predicted [14]. So, for example, on repeatedly seeing that the colour *red* is consistently followed by a food item at location *A*, and that the colour *blue* is consistently followed by a food item at location *B*, a participant learns on subsequent colour events to predict the location of the food item. Essentially, then, associative learning is learning a particular function from a set of cues (e.g., colours) to a set of targets (e.g., locations). An advantage of a functional characterization of associative learning, like the one just given, is that it does not presuppose a particular (e.g., associative strength [15] or propositional [16]) theory of associative learning processes [17].

Despite this seemingly straightforward definition, many factors influence the efficacy of associative learning, such as learning predispositions (i.e. learning biases), temporal contingencies, and cue/target relations, hence the vast literature on this topic [13]. Indeed, whether or not associative learning is mediated by change in associative strength or propositional inference is still actively debated (see [15, 16]). A crucial feature that appears to promote inference driven learning is whether the items being associated can be interpreted in terms of cause-effect relationships [18]. Reasoning takes time, so time pressure promotes alternative associative strength-based learning [19], which aligns with the general *Type 1/Type 2* cognitive process distinction in which timing is a crucial feature [20, 21]. Hence, for the purpose of identifying a paradox for classical theory, we focus on the learning of paired associates whose relationships are meaningless, beyond being predictive (see, e.g., [19, 22, 23]). In this sense, associative learning is characterized as a (second-order) function that takes a list of cue-target pairs and returns a (first-order) function that is a map from a set of cues to a set of targets.

As this set-based, functional characterization suggests, only element (cue) identity is relevant to an (*elemental*) associative process. Cues typically have additional (internal) structure, e.g., words are composed of letters, pictures are composed of pixels, but this structure is not utilized for the computation. This distinction is important, otherwise any input-output map can be regarded as association, a position that we do not support [24]. Clearly, then, such associative processes (first-order functions) cannot support first-order systematicity where the common structural relations are the relations between cue constituents, because these associative processes (by definition) do not make use of internal cue structure. Similar considerations apply to external cue structure, e.g., topological (neighbourhood) and similarity (metric) relations between other cues. Such relations are, of course, important to other kinds of associative and non-associative processes. Our point here is that even in the absence of first-order structure-sensitive processes, there still exists a form of second-order systematicity, which we characterize next.

Our characterization of second-order systematicity of associative learning parallels the usual notion of first-order systematicity. Given that associative learning capacities are second-order functions, as characterized earlier, second-order systematicity of associative learning pertains to the structural relations between such (second-order) functions. In functional terms, second-order systematicity of associative learning is having associative learning capacity (i.e. second-order function) *F*_1_ if and only if having structurally related associative learning capacity *F*_2_, where *F*_*i*_ returns first-order associative capacity *f*_*i*_. Typical examples follow from rote-learning procedures: participants learn a set of cue-target pairs (e.g., a map from a set of four alphabetic characters to a set of four shapes) to some criterion, say, correct target response for a test block of four cues. This procedure is repeated for a different set of cue-target pairs, involving the same classes of cues and targets, or other classes (e.g., a map from a set of four colours to a set of four words). One exhibits a second-order systematicity of associative learning property when one can learn the first map if and only if one can learn the second map. There is no first-order systematicity property here, because the maps share no structural relations among the respective cues.

Clearly, humans exhibit this second-order systematicity property as evidenced by the numerous studies on *learning transfer* effects, also called *learning to learn* (see, e.g., [22, 25], and the cited studies therein). The aim of these studies is to investigate what factors influence the number of learning trials to criterion for each list. Learning the first list typically takes the greatest number of trials. The number of trials required to learn subsequent lists varies depending on, for example, whether they involved the same class of cues/targets as the first list [22], or a repairing of the same set of cues and targets [25]. For our purpose, the relevant data from these studies is that (in general) you do not find participants who can learn one list of paired associates, but not another list. Thus, paired associate learning is a legitimate instance of a second-order systematicity property of human cognition.

An important caveat to this property concerns the learning of *configural* associations, i.e., where outcomes are only predicted by conjunctions of cues, not single cues (elemental association). In terms of list learning, the [AB, CD] condition (i.e., no cue/target is repeated within or between lists) can be considered as elemental association, because every (first list A, or second list C) cue is predictive of every (first list B, or second list D) target independent of (first, second) list context. By contrast, the [AB, ABr] condition (i.e., the second list contains a re-pairing of the cues and targets in the first list) can be considered as configural association, because only the conjunction of list context and cue predicts each outcome. List learning capacity in the [AB, ABr] condition undergoes protracted development from the age of seven [23]. This result is consistent with earlier findings showing that older, but not younger children (around the age of 4.5 years) succeed at *configural discrimination* (respond to A in context 1, but B is context 2) and *transverse patterning* (respond to A in the presence B, B in the presence of C, and C in the presence of A), whose outcomes are only predictive by cue/context conjunction [26] (see also [27]). Elemental and configural associations are distinguished by their *representational rank* [24]. The importance of this difference is that associative learning is not simply one (degenerate) equivalence class of capacities. Just as we can have various schemas for first-order systematicity, we can have various schemas for second-order systematicity of associative learning.

The relationship between first and second-order levels of cognitive capacities accords with the usual treatment of higher-order functions in mathematics and computer science. To wit, a second-order function, e.g., *insert*, can take a first-order function, e.g., addition (+), and return a first-order function, in this case, *sum*: for instance, *insert*(+)[1, 2, 3] = 1 + 2 + 3 = 6. Applying *insert* to the first-order function *lesser* (⊲), i.e., the lesser number (e.g., 4 ⊲ 2 = 2), yields the first-order function *least*: for instance, *insert*(⊲)[4, 2, 6] = 4 ⊲ 2 ⊲ 6 = 2. First-order functions *sum* and *least* are related via the common second-order function, *insert*. Moreover, this relationship between second and first-order functions is analogous to the usual understanding of the relationship between composite representations and their constituents for (first-order) cognitive capacities, as in the (first-order) relation *John loves Mary* and its (zeroth-order) constituents *John* and *Mary*.

Note that second-order systematicity, as the higher-order extension of (first-order) systematicity just described, is not the same notion as *weak/strong systematicity* [12] that was defined as a (learning) criterion to assess whether a connectionist model possessed the “systematicity” property. Weak/strong systematicity is a generalization (prediction) criterion, i.e., given certain kinds of training (learning) examples, where the model is trained to respond with target outputs given corresponding inputs, correctly predict the target responses for certain kinds of testing (unseen) inputs. As such, weak/strong systematicity pertains to the learning of a first-order systematicity property, i.e., acquisition of capacity *c*_1_, by learning on a subset of training examples, implies capacity *c*_2_, as evaluated by a subset of testing examples. Also, recall that (first-order) systematicity [1, 6] is not specifically a generalizability property. Rather, it is an equivalence property: if a cognizer has cognitive capacity *c*_1_, whether or not having this capacity is afforded by some learning/developmental, instructional, or genetically endowed process, then the cognizer also has cognitive capacity *c*_2_. Analogously, for second-order systematicity, if a cognizer has the ability to learn cognitive capacity *c*_1_, whether or not having this capacity is afforded by some learning to learn, teaching to learn, or genetically endowed learning process, then the cognizer has the ability to learning cognitive capacity *c*_2_. In short, weak/strong systematicity concerns the learning of a (first-order) systematicity property, whereas second-order systematicity concerns the systematicity of particular learning capacities (see also Discussion).

Note, further, that second-order systematicity involves two subtypes: (1) second-order systematicity without an accompanying first-order systematicity property, e.g., systematic learning of associations, which is the focus of this paper; and (2) second-order systematicity with an accompanying first-order systematicity property, e.g., systematic learning of natural languages, which we do not explicitly address here. We focus on the first subtype, because it is the simplest example that highlights the need to address a second level of systematicity. Naturally, we acknowledge that the second subtype is also important. This second subtype is discussed elsewhere [28].

### Outline

Perhaps unsurprisingly then, our explanation for second-order systematicity can be characterized as a second-order version of our earlier explanation for (first-order) systematicity (see [10]). In the next section, we argue that the systematic learning of associations presents a paradox for classical theory, because the learned representations share no structural relations between their constituents; more to the point, because there need not be any constituent representations in such cases. In the two sections that follow, we propose a resolution and corresponding model, which follow from our category theory explanation for systematicity based on universal constructions [10]. The current work generalizes the (first-order) notion of shared structural relations between constituent representations to the (second-order) notion of shared structural relations between constituent processes, or second-order relations. The implications of this result are discussed in the last section. The supporting theoretical details are given in the supplementary texts, S1, S2 and S3 Texts.

## Classical compositionality and canonicity

Classical compositionality is the notion that representations and processes pertaining to complex entities, i.e., entities that are constituted of other entities, are composed of representations and processes corresponding to the constituent entities in a *structurally consistent* way. For the *above* example, there is a representation of triangle, a representation of square, and the structural relationship between those two representations corresponds to the spatial relationship of the triangle and square. By virtue of the consistency of this correspondence across instances of object pairs, a capacity to pick out the “top” representation for triangle above square implies the capacity to pick out the top representation for square above triangle, because they are one and the same process.

We can illustrate the way a classical system supports a systematic capacity for representing pairs of items by the following set of production rules (or, grammar), with the relation symbol omitted for simplicity.

$$\begin{array}{ccc} G1:P & \to & S\;T\\ S & \to & △|□\\ T & \to & △|□ \end{array}$$

where | indicates alternative possible expansions of a symbol. Given the start symbol *P*, the system continues to expand non-terminal symbols, in this example, *P*, *S* and *T*, with matching production rules until only terminal symbols remain, which in this example are the symbols △ and □. This set of rules generates all four possible combinations of triangles and squares.

Not all structurally consistent correspondences between representations and the entities being represented support systematicity. To illustrate with the *above* example, suppose we represent object pairs consisting of triangles or squares with symbols △ for triangle, □ for square, and *S* for symbols △ or □, and pairs of objects as the (concatenated) symbols △ *S*, or *S* △. We have the following set of production rules.

$$\begin{array}{ccc} G2:P & \to & \;△S|S\;△\\ S & \to & △|□. \end{array}$$

This alternative system has the capacity to represent triangle above triangle, triangle above square, and square above triangle, but not square above square. Thus, this schema fails to support systematicity in this case.

To rule out grammars such as G2, classical compositionality asserts only the “canonical” grammars, i.e., grammars that support systematicity [6]. Yet, this assertion appears to be ad hoc [3] without some independent principle for determining such constructions. We argue (next) that canonicity leads to a paradox of sorts for the classical approach.

### The paradox of systematic learned associations

A systematic capacity for learning associations appears to be problematic for classical compositionality, since it involves simple associative processes, which were rejected as the basis for a theory of cognitive architecture [1]. Note that this example of systematic learned associations does not vindicate associativism as a viable framework for theories of (first-order) systematicity. Of course, we do find people who know one fact, say, *the first day of the Japanese financial year is April 1st*, without knowing the other fact, *the atomic number of carbon is 6*. Rather, associative theories do not provide a satisfactory explanation for first-order systematicity, because they are equivocal on indivisible (first-order) capacities [1]. The difficulty for the classical explanation is that on the one hand the (canonical) classical constructions are supposed to be just the ones that support systematicity [6], which in this example are associations. Yet, on the other hand, (non-classical) association-based systems fail to pick out the clusters of cognitive capacities that are organized around a common structure [1]. How can the requisite canonical constructions be simultaneously symbolic and non-symbolic (association-based)?

The solution to this apparent paradox appears straightforward: extend the classical explanation to explicitly include a notion of second-order (classical) compositionality. In the context of a set of production rules, or grammar, second-order compositionality is a (second-order) production (or, grammar) that produces a (first-order) production (grammar). For example, with regard to learned associations, we define *rlearn* (recursively learn) to be a second-order production that is given a list of pairs of items to be associated, *pairlist*, and a first-order production, *associate*, which produces the items associated with the given cue, and returns a new first-order production. Informally, *rlearn* is defined as:

$$\begin{array}{c} \mathrm{rlearn}:\{\begin{array}{cc} \left(\mathrm{emptylist},\mathrm{associate}\right) & \mapsto \mathrm{associate};\\ \left(\mathrm{pairlist},\mathrm{associate}\right) & \mapsto \mathrm{rlearn}({\mathrm{pairlist}}^{\prime },\mathrm{update}\left(\mathrm{firstpair},\mathrm{associate}\right)) \end{array} \end{array}$$

where *emptylist* is the empty list of pairs of associated items, *firstpair* is the first pair in the list, *pairlist*′ is the list that remains after removing *firstpair* from *pairlist*, and *update* adds a new pair to the associate production, which may involve adding a new association, or modifying the strength of an existing one. For brevity, *firstpair* is implicitly obtained from *pairlist* by a process that returns the head of a list. An expression of the form *f* : *x* ↦ *y* indicates that the application of function *f* to argument *x* yields the result *y*.

An alternative, non-recursive, form of associative learning is defined iteratively. We define *ilearn* (iteratively learn), which also takes a list of pairs and an associate production and returns a new associate production, as follows:

$$\begin{array}{ccc} \mathrm{ilearn}: & & \\ & \mathrm{while}\;\neg \mathrm{isemptylist}(\mathrm{pairlist})\;\mathrm{do}\\ & \;\mathrm{update}(\mathrm{firstpair},\mathrm{associate})\to \mathrm{associate};\\ & \;\mathrm{tail}(\mathrm{pairlist})\to \mathrm{pairlist}\\ & \mathrm{end} \end{array}$$

where *isemptylist* returns true if *pairlist* is empty, else false, and *tail* returns the list with the first item removed.

Despite the shared variables and subprocess (e.g., *update*), the two versions do not share common relations among their respective constituents. So, in the case that one cognitive capacity is learned (recursively) by *rlearn* and the other cognitive capacity is learned (iteratively) by *ilearn*, there is no (classical) basis for second-order systematicity. Thus, second-order classical compositionality does not necessitate the kind of shared relations between constituent representations that are supposed to explain (second-order) systematicity.

Note, further, that restriction to recursion does not necessarily help, because there may still be (arbitrary) choices to be made, none of which may be satisfactory. For instance, there is a well known tradeoff for list-based recursion that depends on whether lists are processed from the left (head-first), or from the right (tail-first): head-first recursion avoids having to maintain the entire list in memory, but is only applicable when the functions that assemble the intermediate results, e.g., *update*, are associative; tail-first recursion applies to all (associative and non-associative) functions, but requires decomposing the entire list before reassembling results [29]. The production *rlearn* is right recursive. In the current context, head-first recursion lacks systematicity; tail-first recursion lacks credibility in that learning cannot proceed until all examples have been seen. In other words, tail-first recursion does not admit incremental (“on-line”) learning.

The general problem for a supposed (second-order) classical compositionality approach to second-order systematicity is essentially the same problem that arises for the classical approach to first-order systematicity, which is the ad hoc nature of the canonicity assumption. Certainly, a second-order classical compositional system can be devised to address particular instances of second-order systematicity. And, certainly, a second-order classical compositional system can be devised that does not support second-order systematicity. An analogous appeal to canonical second-order classical compositionality does not help, analogously, because there is no characterization of what necessitates just the canonical classical compositions beyond whatever second-order classical composition fits the data, which is characteristically ad hoc [3].

Our diagnosis of the problem afflicting the classical approach is that the focus of compositionality is on the underlying representations and their structure, or lack thereof, rather than the (structure of the) processes that build those representations. As will be apparent shortly, this shift is what one observes from a categorical perspective, where the emphasis is on the morphisms and their compositions [10].

## Categorical compositionality and universality

Much of the systematicity debate has focused on the implementation aspect, i.e., whether or not connectionist claims of (functional) compositionality in support of systematicity are, in fact, just a particular implementation of classical symbolic representation [1, 7, 8, 30]. Category theory [9, 31] is sometimes called a *theory* of structure, which is suggestive of an approach to systematicity that can circumvent implementations issues, and return focus to the central problem of explaining systematicity without ad hoc assumptions. An essential difference between category theory and other formal methods is a shift from the objects or elements of a domain of interest as the primary focus of attention to the relations, or transformations between those objects, which are called *morphisms*. This focus on morphisms affords a unified explanation, based on universal constructions, for second-order systematicity of learned associations that avoids the problem with the classical approach that we just mentioned.

### Categories, functors and universal constructions

Our category-theoretic explanation of systematicity says that underlying every collection of systematically related cognitive capacities is a *universal construction* of some kind [10, 32–34]. To compare this claim with the classical one, we first need to introduce the concepts of *category*, *functor* and *universal construction* (*universal morphism*) in the context of cognition. Further details are given in S1 Text.

Suppose a cognitive system consists of sets of cognitive states, cognitive processes (functions) for transforming cognitive states to new cognitive states, and some means of composing cognitive processes to form other cognitive processes. Processes are composed by making the output of the first process the input to the second process. This basic arrangement can be modeled in terms of a *category*, which consists of a collection of *objects*, a collection of “relations”, or processes between objects called *morphisms*, *maps*, or *arrows*, and an operation for composing morphisms, called *composition* and denoted ∘, that together satisfy certain requirements [9]. For example, a cognitive system could be modeled in the category **Set**, which has sets for objects, functions for morphisms, and composition is composition of functions, assuming that the aspects of the cognitive system being modeled satisfy the criteria for being a category, which are illustrated later.

From this point on, we introduce notation to facilitate descriptions of additional category theory concepts used to address systematicity. A morphism *f* from an object *A* to an object *B* is written *f* : *A* → *B*, where *A* is called the *domain* and *B* the *codomain* of *f*. A morphism *f* : *A* → *B* composed with a morphism *g* : *B* → *C* is the composite morphism *g* ∘ *f* : *A* → *C*. The *identity morphism* associated with an object *A* is written 1_*A*_ : *A* → *A*. Composing a morphism *f* : *A* → *B* with the identity morphism 1_*A*_ or 1_*B*_ results in *f*, i.e., 1_*B*_ ∘ *f* = *f* = *f* ∘ 1_*A*_, and composition is not affected by order of evaluation, i.e., *h* ∘ (*g* ∘ *f*) = (*h* ∘ *g*) ∘ *f*. In the case where the objects are sets and the morphisms are functions, *f* : *A* → *B*, the mapping of each element *a* in *A* to an element *b* in *B* is sometimes written *f* : *a* ↦ *b*, or *f*(*a*) = *b*.

The choice of category will depend on what aspect of cognition is under investigation. When the details of internal states and processes are unknown or not relevant, we may choose an abstract category where the nature of the objects and morphisms is unspecified or without internal structure. In other situations, we may also want to model the internal structure of cognitive states and processes. For instance, networks of associations can be modeled as *directed graphs*, where each graph consists of a set of nodes (the associates) and a set of edges (the strengths of association). Each graph is an object in the category of (directed) graphs, **Grph**, whose morphisms are *graph homomorphisms*, which preserve graph structure, i.e., a graph homomorphism consists of two maps, a map for nodes and a map for edges, such that the source and target nodes of each edge map to the source and target nodes of the mapped edge.

A cognitive system can also be considered as a collection of subsystems, in which case we require a way of modeling the relations between subsystems. If we model subsystems as categories, then an appropriate way to model relationships between subsystems is with morphisms between categories, called *functors*. *Endofunctors* are functors from a category to the same category, which are used to model recursive processes. Categories are “generalized” graphs, in that every category is a graph with a loop at each node and an edge for each connected path. This view facilitates an understanding of functor as a generalized graph homomorphism, or category homomorphism. A category consists of a collection of objects and a collection of morphisms. Hence, a functor consists of a map from objects and a map from morphisms in the domain category to (respectively) objects and morphisms in the codomain category. Thus if *F* : **C** → **D** is a functor and *f* : *A* → *B* is a morphism in **C**, then *F*(*f*) : *F*(*A*)→*F*(*B*) is a morphism in **D**. The morphism component of a functor *F* : **C** → **D** must preserve identities, i.e., *F*(1_*A*_) = 1_*F*(*A*)_, and composition, i.e., *F*(*g* ∘ *f*) = *F*(*g*) ∘ *F*(*f*), cf. graph homomorphism.

The formal, categorical notion of universal construction (universal morphism) is central to our explanation of systematicity. Subsystems provide information commonly required by other subsystems. For example, short-term memory can be thought of as a subsystem for maintaining information about a target item that is required by an executive function for comparison with items currently in the field of view in, say, a visual search task, or a delayed match-to-sample task. Such information is only useful insofar as the structures required by the receiving system are preserved when communicated by the sending system, and in a format that is accessible to the receiver. As we have already seen, a functor models the structure-preserving process. A universal morphism models the situation that this information is universally (systematically) accessible to the receiver.

Category theory constructions, including universal morphisms, typically come to two forms that are based on the directions of the arrows that constitute a particular construction. The relationship between such constructions is called *dual*. For instance, *initial object* is an object that has an arrow from it to every object in the ambient category. *Terminal object*, dual to initial object, is an object that has an arrow to it from every object in the ambient category. Dual constructions are often labeled with the prefix “co”. So, the concepts of *coalgebra* and *corecursion*, introduced in the next section and central to our categorical approach to second-order systematicity, are the dual constructions of the more familiar notions of algebra and recursion.

### Coalgebras and corecursion

Category theory provides a systematic treatment of corecursion in the form of coalgebras and anamorphisms, which form the basis for our categorical model of associative learning. For comparison, the more familiar (dual) notion of recursion, and its category theory treatment, is given in S2 Text. Here, we provide several simple examples of anamorphisms as a conceptual guide to the theory (see S1 Text), and our subsequent model.

Repeating an item *n* number of times is realized as the anamorphism, $\mathrm{unfold}(0?\to I_{*},\langle 1,\mathrm{dec}\rangle ):N\to L$, where 0? tests whether a number is zero, $I_{*}$ is the constant function returning the unnamed element *, 1 is the constant function returning 1, *dec* decrements a number by 1, and so 〈1, *dec*〉 is the *product function* *n* ↦ (1, *n* − 1)—in general, 〈*f*, *g*〉 : *x* ↦ (*f*(*x*), *g*(*x*)). See S1 Text (Diagram 9) for comparison. Using ⋅ to denote *prepend* (also called *cons*), i.e., *h* ⋅ *t* prepends (head) element *h* to (tail) list *t* returning the list with *h* as the first element and *t* as the rest of the list, and [] to denote the empty list, we have for instance:

$$\begin{array}{cc} & \mathrm{unfold}(0?\to I_{*},\left\langle 1,\mathrm{dec}\right\rangle )\left(3\right)\\ & \;=1\cdot \mathrm{unfold}(0?\to I_{*},\left\langle 1,\mathrm{dec}\right\rangle )\left(2\right)\\ & \;=1\cdot 1\cdot \mathrm{unfold}(0?\to I_{*},\left\langle 1,\mathrm{dec}\right\rangle )\left(1\right)\\ & \;=1\cdot 1\cdot 1\cdot \mathrm{unfold}(0?\to I_{*},\left\langle 1,\mathrm{dec}\right\rangle )\left(0\right)\\ & \;=1\cdot 1\cdot 1\cdot [\;]\\ & \;=[1,1,1]. \end{array}$$

That is a list of three 1’s. Notice that the anamorphism just given is a state-less (or, memory-less) computation. To count items, we must retain the number of previously counted items. For example, $\mathrm{unfold}\left(e?\to I_{*},\langle \mathrm{incl},\mathrm{tailr}\rangle \right):N\times L_{X}\to L_{N}$ takes the number of items counted so far, n∈N and a list *l* ∈ *L*_*X*_ of elements from *X*, and returns the progressive count of list items $c\in L_{N}$. In this example, the conditional *e*? tests for an empty list (at the second component of a given pair), i.e., no more items to be counted, and terminates the count when the list of remaining items is empty, via $I_{*}$, or increments the count and removes the counted item from the list, via product function 〈*incl*, *tailr*〉. The function *incl* : (*n*, *l*) ↦ *n* + 1 increments the counter (left component) and ignores the list; the function *tailr* : (*n*, *h* ⋅ *t*) ↦ (*n* + 1, *t*) maintains the new count and removes the counted item from the list of items to be counted. Compare S1 Text (Diagram 9): object *A* is now the set of natural numbers N, and *X* is the Cartesian product $N\times L_{X}$ of the natural numbers with the set of lists of elements from a set *X*. For instance,

$$\begin{array}{cc} & \mathrm{unfold}\left(e?\to I_{*},\left\langle \mathrm{incl},\mathrm{tailr}\right\rangle \right)\left(0,\left[a,b,c\right]\right)\\ & \;=1\cdot \mathrm{unfold}\left(e?\to I_{*},\left\langle \mathrm{incl},\mathrm{tailr}\right\rangle \right)\left(1,\left[b,c\right]\right)\\ & \;=1\cdot 2\cdot \mathrm{unfold}\left(e?\to I_{*},\left\langle \mathrm{incl},\mathrm{tailr}\right\rangle \right)\left(2,\left[c\right]\right)\\ & \;=1\cdot 2\cdot 3\cdot \mathrm{unfold}\left(e?\to I_{*},\left\langle \mathrm{incl},\mathrm{tailr}\right\rangle \right)\left(3,\left[\;\right]\right)\\ & \;=1\cdot 2\cdot 3\cdot [\;]\\ & \;=[1,2,3]. \end{array}$$

Notice, further, that this count anamorphism returns a list of counts, not a single count. The elements of such output (likewise, input) lists are commonly interpreted as being indexed by steps in time for corecursive models of data streams, i.e., infinite lists [35]. We invoke a similar temporal interpretation of lists for our learning model.

## Corecursion model of associative learning

Suppose we regard learning as a kind of recursive process in the sense of taking the current cognitive state and an input and returning the next cognitive state, which in category-theoretic terms involves an endofunctor. Category theory provides a unified (systematic) treatment of recursion [36, 37]. Associative learning can be regarded as a form of learning as recursion where the state of the cognitive system includes the strengths of associative links between representations, or more generally a network of associations, and the inputs are perceived co-occurrences of entities. Notice that although the associated representations may have classical structure, there need not be any systematic relationship between such structures involved in the learning of different associations, as our memorization of facts example illustrated. We have already provided a category theory treatment of recursive cognitive capacities, generally [33]. The universal morphism in such situations captures the common recursive aspect of the process. Technically, the universal morphism in these cases is either: an *initial algebra* in a category of algebras on an endofunctor and each recursive process is modeled as the unique (*cata*)morphism from the initial algebra; or, dually, a *final coalgebra* in a category of *coalgebras* on an endofunctor and each (*co*)recursive process is modeled as the unique (*ana*)morphism to the final coalgebra. We develop our model in two steps for expository purposes. The first step treats the association network as an explicit input. This approach is simpler, but unrealistic since memory is treated as external input. The second step treats memory as internal using *adjoint anamorphisms* (*adjoint unfolds*), explained later. Again, for comparison, a (dual) recursive formulation is given in S2 Text, which also provides motivation for our coalgebraic approach.

### Network state as external input

The capacity for learning associations is modeled as a function from a list of pairs (associates) to an association network. Recall, from the counting example, that a simple anamorphism does not maintain a state, and so does not suffice as an associative learning model, since previous associations are lost. A memory is maintained by passing the results of earlier items as an explicit input to the model. Accordingly, associative learning is modeled as a function from a list of pairs and an association network to an updated association network. The anamorphism (model) is indicated by the diagram

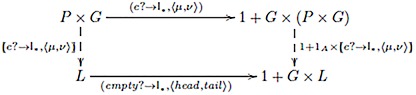

where:

- *P* is a set of lists of pairs of associated items;
- *G* is the set of (labeled) directed graphs (association networks), where each graph *g* ∈ *G* is a pair (*E*, *V*) consisting of a set of edges *E* and a set of vertices *V*, and each edge is a triple (*s*, *σ*, *t*), where *s* and *t* are the source and target vertices and *σ* is the strength of association; hence
- *L* is the set of lists of association networks of type *G*;
- *e*? : *P* × *G* → {*False*, *True*} tests for an empty list of associates, and *empty*? : *L* → {*False*, *True*} tests for an empty list of graphs;
- *μ* : *P* × *G* → *G* is a function that merges the current pair of associated elements with the current association network, returning an association network; and
- *ν* : *P* × *G* → *P* × *G* is the next state function that returns the list of remaining pairs, and the merged association network, i.e., *ν* = 〈*τ*, *μ*〉, where *τ* : *P* × *G* → *P* returns the tail of the pairs list, which ignores the association network.

The merge function, *μ*, can be instantiated in many ways depending on the specific form of learning being modeled. For instance, the strength of association *σ* for a given pair of associates could be updated to $\frac{1+\sigma }{2}$ on each co-occurrence, in which case associative strength increases monotonically with number of co-occurrences from *σ*_0_ (initial strength on first occurrence) to 1. Other considerations are straightforwardly included, such as decay and normalization terms (see S3 Text, for examples).

A simple example suffices to illustrate the mechanism. The anamorphism given by the above diagram is relabeled *m*_*ext*_, the model with external memory. Suppose the initial list of pairs: [(bread, butter), (knife, fork), (knife, butter)]. The initial state of the association network is set to the empty graph *e*. We denote pair and network lists at time *t* as *p*_*t*_ and *g*_*t*_, respectively. Hence, the initial pair list *p*_0_ contains three pairs, and the initial network *g*_0_ = *e*. The first step in time is *m*_*ext*_(*p*_0_, *g*_0_) = *g*_1_ ⋅ *m*_*ext*_(*p*_1_, *g*_1_), where *g*_1_ is the association network containing the single edge *σ*_1_ : bread → butter (i.e., an association from bread to butter with strength of association *σ*_1_), and *p*_1_ is the pairs list [(knife, fork), (knife, butter)]. This process continues corecursively to obtain *g*_1_ ⋅ *g*_2_ ⋅ *g*_3_ ⋅ *m*_*ext*_(*p*_3_, *g*_3_) at which point the model returns the empty list (of networks) and terminates with the list [*g*_1_, *g*_2_, *g*_3_]. That is the evolution of association networks over time steps, with *g*_3_ being the final network state indicated by the following diagram:

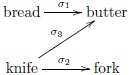

In the case of semantic systematicity, which entails understanding the meaning of associated constituents, *G* is a set of semantic association graphs; in the case of syntactic systematicity, where constituents (e.g., bread) are understood as just a sequence of characters, *G* is a set of symbol (string) association graphs.

An important feature of the anamorphism approach, in contrast to a catamorphism approach, is that the computation at each (time) step proceeds independently of the remaining steps. For example, the first item of the list *g*_1_ ⋅ *m*_*ext*_(*p*_1_, *g*_1_), i.e., *g*_1_, is not affected by the computation of the rest of the list. This property of anamorphisms justifies the temporal interpretation of lists. Effectively, then, there is only one association graph produced by the model, whose state is indexed by time step *t*, i.e., the network *g*_*t*_ in the list *g*_0_ ⋯ *g*_*t*_ ⋅ *m*_*ext*_(*p*_*t*_, *g*_*t*_).

### Network state as internal memory

The previous model depends on treating network state as a kind of external memory. The theory of adjoint catamorphisms and anamorphisms—adjoint folds and unfolds [38]—allows us to treat network state as internal to the model. We make use of the product-exponential adjoint introduced in S1 Text. This construction effectively provides a universal means of transforming the external state map into an internal state map, as indicated by the following diagram (highlighting the bijection aspect of this adjunction):

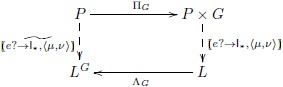

The internal model, which we denote *m*_*int*_, is the *exponential transpose* of the external model *m*_*ext*_. That is, $m_{\mathrm{int}}=\tilde{m_{\mathrm{ext}}}$. See S1 Text (Example 6) for further details.

A final coalgebra is a universal construction. Thus, we have shown that the (second-order) systematicity of associative learning follows from the same category theoretical principles as other (first-order) forms of systematicity. The formal connection between final morphism and final coalgebra (dually, initial morphism and initial algebra) is given in the supplementary texts, S1 and S2 Texts.

### Second-order systematicity of paired associate learning

With the coalgebraic model in place, we return to the example of second-order systematicity of paired associate learning, which we mentioned in the Introduction. For concreteness, suppose we have a set of four word-shape associations that are specified by the following function: *ws*_1_ : *Word*_1_ → *Shape*_1_; bell ↦ □, kite ↦ △, tent ↦ ♡, yacht ↦ ◇. The set of pair lists, *P*_1_, contains the list of pairs used to learn the associations, e.g., [(bell, □), (kite, △), …]. List type, *G*_*WS*_, is the subset of all possible directed graphs, as defined earlier, see the first diagram (“Network state as external input” section) and subsequent text, whose vertices are taken from the set of representable words and shapes. The anamorphism for learning this association is the function ${\mathrm{WS}}_{1}:P_{1}\to L^{G_{\mathrm{WS}}}$, cf. the diagram in the section “Network state as internal memory”. Suppose we have a new set of word-shape associations specified by the function, *ws*_2_ : *Word*_2_ → *Shape*_2_; goat ↦ ▴, lion ↦ ◾, mule ↦ ♣, toad ↦ ♠. The set of list pairs, *P*_2_, contains the training list [(toad, ♠), (lion, ◾), …]. The second-order systematicity property is that one can learn *ws*_1_ if and only if one can learn *ws*_2_. The universal mapping property guarantees the existence of associative learning function ${\mathrm{WS}}_{2}:P_{2}\to L^{G_{\mathrm{WS}}}$. Thus, we have second-order systematicity of paired associate learning.

As mentioned in the Introduction, both first-order and second-order systematicity assume that the constituents (e.g., words and shapes) are representable. In the same manner, the capacity for configural association assumes that conjunctions of cues are representable. Suppose we have a set of configural associations specified by the function *ca* : *Colour* × *Shape* → *Response*, which has the following mappings: (black, square) ↦ +, (black, triangle) ↦ −, (white, square) ↦ −, (white, triangle) ↦ +. In this case, list type is the subset of all possible directed graphs, *G*_*C*_, whose vertices include the set of representable conjunctions, e.g., ColourShape={◾,▴,□,△}. By contrast, the list type for elemental associations is the subset of directed graphs, *G*_*E*_, that does not include conjunctions. Thus, we have a different universal morphism (final coalgebra) for configural association. Just like first-order systematicity, where the capacity for *square above triangle* does not imply a capacity for *John loves Mary*, a second-order capacity to learn elemental associations does not imply a capacity to learn configural associations: the *above* versus *loves* capacities involve different relational schemas, the elemental versus configural capacities involve different associative schemas.

### Empirical tests for universal constructions

In principle, since every universal construction is either an initial or terminal object in the corresponding comma category [9], a succinct schema for empirically testing the universal construction account of systematicity follows. The claim is that a pair of systematically related cognitive capacities *c*_1_ and *c*_2_ is explained by a pair of arrows *u*_1_ : 0 → *A*_1_ and *u*_2_ : 0 → *A*_2_, which are related by an initial object, 0; or, dually, a pair of arrows *v*_1_ : *A*_1_ → 1 and *v*_2_ : *A*_2_ → 1, which are related by a terminal object, 1. Hence, a test for a universal construction as an initial object has three parts:

1. a test for the underlying arrow *u*_1_ : 0 → *A*_1_;
2. a test for the underlying object *A*_2_; and
3. a test for the underlying arrow *u*_2_ : 0 → *A*_2_.

So, evidence for 1 and 2, but not 3 counts against the universal construction account of systematicity. The test for having *u*_2_ implies having *u*_1_ is essentially the same, but with the roles of *u*_1_ and *u*_2_ interchanged. The test for universal construction as a terminal object case proceeds analogously. In practice, since these objects will have their own internal structure, the corresponding tests at each step will also involve multiple interceding steps.

In general cognitive terms, an anamorphism to a terminal (final) coalgebra builds up some internal representation, e.g., parse tree; a catamorphism from an initial algebra collapses some internal representation to some summary value, e.g., whether a sentence is grammatical or not. Accordingly, the names for these kinds of morphisms derive from Greek prepositions, *ανα* meaning “upwards”, and *κατα* meaning “downwards” [39]. Note that in this example, the (co)inductive structure is a tree. See [33] for further examples related to cognition, and [40] for examples related to computational (operational versus denotational) semantics.

## Discussion

The classical explanation, as commonly construed, is a “first-order” explanation of systematicity, vis-a-vis the tokening principle, whereas systematicity of associative learning is a second-order property. That is, classical theory explains systematicity in terms of common first-order processes that token constituent representations. However, in general, the representations of learnable associations may have no common constituents at all. For second-order systematicity the common constituents are the constituent learning processes that construct associative processes, i.e., common second-order constituents. Put simply, an explanation for second-order systematicity derives from second-order compositionality. Naturally, classical theory admits a notion of second-order compositionality where the common constituent symbols range over processes instead of representations. However, a notion of second-order classical compositionality reintroduces the canonicity assumption, as we have already explained. Moreover, associative strength is a numeric quantity not a symbolic one, so it is unclear how symbols are supposed to explain associative learning.

Category theory resolves this problem via a generalized notion of compositionality: composition of morphisms, which subsumes (first-order) compositionality of representations and (second-order) compositionality of processes, to provide an explanation for both corresponding forms of systematicity. Furthermore, category theory offers a way forward in regard to the classical canonicity assumption, because every universal construction is also the optimal construction in its associated *comma category* [9]. This fact inspired a category theory treatment [41] of systematicity in analogy [42] and its relation to the systematicity property for cognition architecture [1]. Thus, our universal construction approach avoids one of the characteristics of ad hoc assumptions, i.e., unconnectedness, in that the two apparently different kinds of compositionality are in fact two instances of the same construction, that is a universal construction. Moreover, that every universal construction is an optimal construction, i.e. in the sense of having a connection to every object in that category, suggests that they derive from some form of optimization process.

An advantage of category theory is that it provides a principled approach to (co)recursive cognitive capacities. Symbol systems admit arbitrary recursive constructions, but not every recursive formulation is systematic in the sense of being well-defined over all possible inputs. Well-definedness of recursive constructions in the categorical setting depends only on the well-definedness of the given *F*-(co)algebra, since the (unique) existence of an anamorphism (or, catamorphism) is guaranteed by the universal property. Existence and uniqueness were motivations for taking a category theory approach to recursion in the first place (see, e.g., [37]).

The generality of anamorphism may leave some people wondering whether it is too general. In particular, since many species have the capacity for simple associative learning, why then do they not also have the capacity for more advanced forms of learning, such as learning via analogy? Recall that the systematicity problem is at the level of the complex entities, not at the level of their components. For example, the capacity to understand that *John loves cricket* implies the capacity to understand *John loves baseball* given that one understands that *John* refers to a person, and that *cricket* and *baseball* refer to games. The capacity to understand that *John loves cricket* does not imply the capacity to understand *John loves hanafuda* when one does not understand the meaning of *hanafuda*—a Japanese card game—in the case of semantic systematicity. Likewise, we don’t expect a capacity for learning associations to imply a capacity for learning by analogy, because association and analogy involve different kinds of underlying structures [24]; categorically, they involve the existence of different objects (*F*-coalgebras). Rather, we expect that if a subject has the capacity for the underlying (coalgebraic) structures, then a capacity for learning with respect to one kind of structure implies a capacity for learning with respect to the other kind of structure, because they both involve the same form of (co)recursion. That is, they involve the same final coalgebra, and the unique existence of the corresponding anamorphism is guaranteed by the universal property.

### Categorical versus classical theory

Despite the foregoing analysis, some readers may still ponder the essential advance that the categorical explanation offers over the classical one, since symbol systems are routinely developed to treat functions as data for other (higher-order) functions, in the functional programming style. Perhaps ironically, given that category theory has some notoriety for “arbitrary abstraction”, the categorical explanation provides more precise (universality) conditions for the kinds of compositions that yield systematicity as opposed to the kinds of compositions that do not, and importantly these conditions are given in terms of categories and functors, not the classes of systematically related cognitive capacities they are supposed to explain.

There is, of course, overlap between the two explanations, given that they both appeal to some form of compositionality. Classical theory appeals to a notion of compositionality as some form of *relational homomorphism* (see [1], footnote 9): informally, the relations between the constituent (symbolic) representations are mirrored by the relations between the constituents of the entities being represented; more formally, a relational homomorphism is a map *h* from a relation *R* to a relation *S* such that if *a* is related to *b* by *R*, i.e., *aRb*, then *h*(*a*) is related to *h*(*b*) by *S*. Category theory also includes relational homomorphism, and (as we have already seen) other kinds of homomorphisms, including homomorphisms as morphisms between objects (e.g., graphs), functors between categories, natural transformations between images of functors, and adjunctions relating functors, considered as homomorphisms between homomorphisms.

From a classical perspective, categorical compositionality may be seen as a (generalized) version of the classical tokening principle, in the sense that the instantiation of every compositional arrow (e.g., *g* ∘ *f*) entails the instantiation of each of its constituent arrows (i.e., *f* and *g*), just as the instantiation of every complex symbolic expression (e.g., “John loves Mary”) entails the instantiation of each of its constituent symbols (i.e., “John”, “loves”, and “Mary”). However, from a category theory perspective, not every composition involves a universal construction, just as not every instance of (generalized) tokening supports systematicity. For this reason, the categorical notion of universal construction is a significant advance over classical and connectionist explanations.

Where the categorical explanation goes significantly beyond the classical one is in the additional axioms that dictate what is a particular construction. The classical criterion of relational homomorphism is too weak, which forces adjoining the canonicity assumption. As the categorical approach makes transparent, not every (relational homo)morphism is a universal (homo)morphism. The problem with the canonicity assumption is that it is unclear what makes a classical construction a canonical one, independent of convention or the criterion that it is whatever construction picks out just the systematically-related cognitive capacities. By contrast, the categorical explanation for universality is specified independently of a particular category or functor, as we have already seen. For this reason, category theory provides a natural explanation that subsumes both first-order and second-order systematicity.

### Tokened versus non-tokened constituents

Related to our distinction between categorical and classical compositionality is the (classical) notion of tokening. Much has been made of distributed vector representations as a potential non-classical (connectionist) version of compositionality that supports systematicity [30, 43]. The basic idea is that distributed representations avoid the tokening principle, because the composition of two vectors need not literally contain the vectors being composed. Contrast, for example, the concatenation of vectors *v*_1_ = (0.1, 0.3, 0.2) and *v*_2_ = (0.7, 0.4, 0.6) yielding the vector *v* = (0.1, 0.3, 0.2, 0.7, 0.4, 0.6)—the inscription of *v* literally entails the inscriptions of *v*_1_ and *v*_2_, against the *outer product* of *v*_1_ and *v*_2_ yielding the matrix, written as the vector *w* = (0.07, 0.04, 0.06, 0.21, 0.12, 0.18, 0.14, 0.08, 0.12)—the inscription of *w* nowhere entails the inscriptions of either *v*_1_ or *v*_2_.

Categorical compositionality also admits this kind of distributed (non-tokening) compositionality. To illustrate, pairs of elements can be represented in a product vector space, which is another kind of universal construction, in the category of vector spaces and linear functions. Localist vector representations can be converted into distributed vector representations via a vector space rotation operation. This distributed representation is also a product, which is isomorphic to the localist one. With a distributed encoding, failure of a single neuron causes partial degradation across many representations; with a local encoding, failure of a single neuron causes complete loss of a single representation. As with the classical case, not just any distributed representations will do. We require those distributed representations with the universal mapping property. Such alternative universal constructions are generally regarded as the “same” in the sense of being *unique up to a unique isomorphism* [9]. Thus, another benefit of category theory is that it clarifies the relationship between these two forms of compositionality: tokened versus non-tokened.

Notice that in our example of paired associate learning the association graphs have both tokened and non-tokened constituents. The tokening of each graph includes the tokening of its vertices, representing the cues and the targets. In contrast, the numeric strength of association, which determines the associate of a given cue, does not include the tokening of each cue-target co-occurrence in the list of training pairs. Note, also, that one can construct a classical tokening version of associative strength by representing cue-target association as a list that contains one element for each experienced co-occurrence of the corresponding cue-target pair. In this case, the associate of a cue can be determined by the edge with greatest list length. The general point here is that tokening is sufficient, but not necessary. What is missing from the classical account (and indeed the connectionist one) is the universal construction component, which provides the sufficient and necessary condition (i.e. the final coalgebra) for second-order systematicity of associative learning.

That there are both tokening and non-tokening ways of realizing associative strength illustrates a further point: even for the relatively simple process of associative learning, which can be straightforwardly implemented in a conventional neural network model (see S3 Text), there is nothing within connectionist theory that ties them together so that one associative learning capacity occurs if and only if the other does. One can also straightforwardly configure a neural network to learn one set of associations without being able to learn the other. This situation echoes the original (first-order) systematicity problem that connectionism faced [1]. The final coalgebra approach says that the systematically related learning capacities derive from a common component process that operates on the same type of network.

### External versus internal structure

A notion of non-tokened constituent may seem mysterious in the light of Fodor and Pylyshyn’s critique [1] of Smolensky’s non-classical approach [8] to systematicity: the essential problem was that non-tokened (i.e. virtual) constituents don’t have causal efficacy. Another way to characterize the difference between the categorical versus classical approach that demystifies this notion is in terms of the difference in focus on external versus internal structure. In a category of graphs, for example, each graph *G* has two kinds of structure: (1) the morphisms that relate *G* to other graphs in the category—external structure, and (2) the edges that relate each vertex *G* to other vertices in *G*—internal structure. Likewise, in a category of vector spaces, each vector space *V* has external structure (linear maps to other vector spaces) and internal structure (inclusion relations between subspaces of *V*). As a universal construction, a product of vector spaces consists of a product vector space (*V* × *W*) and two projections (e.g., *p*_1_ : *V*_1_ × *V*_2_ → *V*_1_). That projection structure is external to the vector space, so either tokening or non-tokening of object constituents (i.e. employing local or distributed vectors) suffice, since both afford domains for the cognitive processes modeled; they are the same up to a unique isomorphism, as mentioned. By this characterization, the problem of second-order systematicity for the classical (first-order) explanation is not so much that tokening poses a paradox, but that tokening (versus non-tokening) of object constituents is irrelevant.

Fodor and Pylyshyn were concerned with the putative claim that one could represent complex entities, in a systematic way, without having to implement classical compositionality, i.e. without also having to “literally” represent the entities constituents. Opponents of the classical position typically interpret “literal” literally, e.g., as in the already discussed difference between local versus distributed representations. However, Fodor and Pylyshyn intended “literal” to refer only to the symbolic level, not the implementation level where symbols may be realized by distributed vector representations. Local and distributed vectors purportedly representing complex entities can be decomposed in arbitrarily many ways, because a vector space can have arbitrarily many bases (i.e., sets of basis vectors) only some of which afford systematic retrieval of the component vectors that correspond to the constituent entities. Fodor and Pylyshyn’s claim was that systematic retrieval is only possible when one stipulates access functions that effectively implement classical compositionality. So, much of the systematicity debate focussed on whether or not connectionism should be regarded as an implementation of classical theory. However, from a category theory perspective, the classical focus on internal structure is doubly misdirected. Not only is the tokened versus non-tokened distinction irrelevant, but the decomposition of symbols (strings) representing complex entities into component symbols (substrings) that systematically represent corresponding constituents also assumes a particular mode of decomposition. There are many ways to split strings, only some of which support the requisite instance of systematicity, hence the auxiliary (ad hoc) canonicity assumption introduced into the classical explanation. What is missing with both tokening and non-tokening schemes is a corresponding notion of universal construction.

We note in passing that this distinction between external versus internal structure calls to mind an explanatory limit in regard to a category theory approach to cognition. Although the collections of many kinds of internally structured objects constitute categories, such objects may not form categories in their own right. Examples of objects with internal structure that are not categories include semigroups (identity is not required), groupoids (composition is not required), and graphs (neither identity nor composition is required). And so, category theory has nothing to say about the *internal* nature of cognitive processes best modeled by these kinds of objects. However, in each case, category theory has potentially something to say about the *external* nature of such cognitive processes, since their collection forms a category.

### Second-order versus weak/strong systematicity

Systematicity [1] pertains to explaining why having capacity *c*_1_ implies having capacity *c*_2_, but not why/how *c*_1_ is obtained/learned in the first place. Weak/strong systematicity [12] also pertains to explaining why/how *c*_1_ is learned, via some training set, in a way that *c*_2_ is also obtained, as demonstrated by performance on some test set. See [44] for an example of acquiring a systematicity property from a realistic training set. So, an explanation for weak/strong systematicity from our category theory approach would entail providing an explanation for the learning of universal constructions. By our coalgebraic approach, learning a universal construction involves constructing a suitable category with a suitable universal arrow. In this situation, we have universal constructions at two levels: at the first level with regard to the constructed graph/category, and at the second level with regard to the category (of coalgebras) that constructs graphs/categories. Put succinctly in the context of learning, second-order systematicity is the systematicity of learning; weak/strong systematicity is the learning of systematicity.

The categorical framework, and more specifically, the coalgebraic approach can be used to develop a system that learns to acquire systematicity through examples. For instance, the input is a list of training pairs, and the coalgebra updates the weights of a neural network via backpropagation. The merge function, *μ*, see the first diagram (“Network state as external input” section) and subsequent text, in this case will include forward and backward propagation components. Note that both forward and backward components can also be defined recursively, i.e., as recursion over a list of weighted layers of neural units, suggesting that the *μ* component itself involves a (co)algebra. Once trained, the generalization performance of the network can be evaluated via testing examples, in the usual way. The performance of the network will depend on the training set, network connectivity, as well as other parameters. So, although the system has the capacity to learn, there may be performance-related reasons why learning may fail in a given situation. For example, the system may fail to learn to be systematic because of the well-known problem of local minima: the state of the system, prior to learning, has weights that lie in the vicinity of a local minimum which can preclude learning the target function. This distinction between capacity (competence) and performance is further discussed in the next section.

### Competence versus performance

Our explanation of systematicity employs the usual competence-performance distinction, following others [1, 3, 6], famously introduced by Chomsky in the context of language. However, see [44] for a treatment of systematicity in terms of performance. Roughly, competence is what one can do under amenable conditions; performance is what one actually does when other extraneous factors are introduced that typically arise in real-world settings. A failure in performance does not necessary imply a failure in competence, especially when the extraneous factors are unrelated to the cognitive capacity of interest. As an extreme example, an obstruction of the visual field does not imply a failure in the capacity to read.

In other situations, additional performance-related factors may indeed play an important role in understanding failures of systematicity. For example, when one considers the cost of learning a universal construction against the benefit it affords, a general question arises: why incur the expense of inducing a reusable component if that component will rarely be reused? We have made tentative steps in explaining failures of systematicity along this line. Each cognitive capacity (arrow) is associated with a resource cost. When the number of related capacities is small, the saving in cognitive resources afforded by reuse may not outweigh the cost associated with inducing the universal construction, hence a failure of systematicity [28]. Further work is needed to develop a category theoretical account of systematicity and its failures.

### Equivalence class versus schema

McLaughlin [6] characterizes instances of systematicity via specific schemas, e.g., “(SG1) Ceteris paribus, a cognizer is able to mentally represent that *aRb* if and only if the cognizer is able to mentally represent that *bRa*.” (see SG1-SG5 on p. 272 of [6]). The phrase “if and only if” denotes a formal equivalence. Equivalence determines an equivalence relation and hence a partition into equivalence classes. Although McLaughlin does not use the phrase “equivalence class” in his paper, the expression “if and only if” means that equivalence classes are tacitly present.

Our use of equivalence relations greatly extends McLaughlin’s use of schemas to characterize examples of systematicity. Note, however, that we are not saying that just any equivalence relation forms the basis for systematicity. Any set can be partitioned into an arbitrary collection of (non-empty) subsets by fiat. For example, the facts “the atomic weight of carbon is 6” and “the beginning of the Japanese financial year is April 1st” can be placed in the same equivalence class, by simply listing the members of each class, implying that they are systematically related, which they are not at the level of those facts. Rather, we are saying that the relevant equivalence relations are the ones that are determined by universal constructions. For the kind of systematicity of associative learning that motivated the current work (i.e., systematic associative learning capacity over unrelated associates), the relevant equivalence relation is at the level of associative processes, which is the final coalgebra, not at the level of the items being associated. This situation is an example of having second order systematicity without having first order systematicity, as mentioned in the Introduction. Other forms of systematic associative learning that also depend on the items being associated are discussed in the “Wide versus narrow systematicity” subsection, later.

The relationship between universal construction, equivalence class and systematicity may itself sound paradoxical given that every collection of equivalence classes, determined by an equivalence relation, can be expressed as a *coequalizer* [45], i.e. another kind of universal construction. However, there is no paradox, because in this situation the coequalizer is derived from an independently given equivalence relation. That is, given an equivalence relation *R* over a set *A*, we obtain the coequalizer of projections *p*_1_, *p*_2_ : *R* → *A* as an assignment of the elements to equivalence classes, *q*_*R*_ : *A* → *R*/*A*, in a minimal way, i.e., every other assignment factors through *q*_*R*_. Since *R* need not be derived by some other universal construction, there is no claim of systematicity, as we would expect.

### Elemental versus configural association

The data with regard to development indicate that both younger and older children can learn elemental associations, but configural assocations are difficult to learn for younger children [26, 27]: the set of elemental associative learning capacities is a subset of the union of the sets of elemental and configural associative learning capacities. This inclusion relationship between capacities seems to violate our characterization of systematicity in terms of (structural) equivalence relations—recall that equivalence classes are disjoint. Note, however, that in category theory every function (and morphism generally) is distinguished (in part) by their (co)domain. So, for example, the function *f* : *A* → *B* and the function *f* *restricted to* *A*′ ⊂ *A*, denoted *f*|_*A*′_ : *A*′ → *B*, are two distinct functions. (If *A* = *A*′ then *f* and *f*|_*A*′_ are the same function.) Inclusion relations between functions are defined analogously. However, our characterization of systematicity is given in terms of structural relations over morphisms (*f* : *A* → *B*) not their mappings (*f* : *a* ↦ *b*, in the case that the relevant morphisms are functions between sets or set-like objects, e.g., groups). Hence, the equivalence classes are indeed disjoint. Put another way, although a capacity for elemental *and* configural associative learning implies a capacity for elemental associative learning, it does not imply a capacity for elemental associative learning *only*. In the same way, more generally, inclusion relations between cognitive capacities do not violate our characterization of systematicity in terms of equivalence classes.

Recall, also, our earlier discussion on the external (categorical) versus internal (classical) perspective on structure. A focus on internal structure in terms of the domains of cognitive processes would violate the very characterization of systematicity that classicists purport to explain, as we have just discussed with regard to elemental versus configural associations.

### Wide versus narrow systematicity

Up to this point, we have appealed to a general capacity in humans to learn or memorize cue-target associations over a wide range of stimuli as examples of second-order systematicity properties. A systematic capacity for learning associations in other species, in contrast, can be more narrowly selective, e.g., rats can learn odour-food associations, but not colour-food or tone-food associations [46]. Selectivity varies not only with stimulus type, but also with response type, e.g., pigeons learn to associate colour with food, but not tone; and learn to associate tone with shock avoidance, but not colour [47]. The kind of selectivity also varies with species, e.g., rats learn to avoid flavoured water, whereas quails learn to avoid coloured water that in both cases was subsequently associated with an induced illness [48]. This difference concerns the scope, not the absence, of a second-order systematicity property, except in the extreme case of one-capacity equivalence classes, whence there is no second-order systematicity property to be explained. How, then, can we reconcile a wide form of systematicity for associative learning in humans with an almost arbitrarily narrow form in other species?

The differences lies with the “type” of the universal morphism. Recall that a universal morphism is a pair (*A*, *ϕ*), see S1 Text for details. Thus, each universal morphism and category associated with that universal morphism, as its initial/terminal object, is indexed by *A*. In the context of (co)recursion, object *A* is the type of the elements that make up each list. As the basis for systematic associative learning, each final coalgebra is indexed by the set *G* of possible association graphs/networks: every anamorphism in the associated category constructs a list of type *G*. So, the extent of *G* determines, in part, the breadth of the corresponding equivalence class of associative learning capacities. For example, the set of networks for rats, *G*_*Rat*_, consists of populations of neurons for representing colours, odours, and foods, and associative connections from odours to foods for learning odour-food associations, but not colours to foods which precludes learning colour-food associations. Hence, there is a coalgebra/anamorphism for learning odour-food associations, but no coalgebra/anamorphism for learning colour-food associations: there is no merge function (connection strength update rule) defined over such networks, because these networks have no such connections to update. In contrast, the set of networks for quails, *G*_*Quail*_, consists of networks with colour-food, but not odour-food connections, which affords the capacity to learn colour-food, but not odour-food associations (see S3 Text for further details). Hence, different species can have differing second-order systematicity properties regarding associative learning.

Within the context of, for example, odour-food associations, one can have a narrow form of second-order systematicity in that one has the capacity to learn to associate with food odour *o*_1_ if and only if one has the capacity to learn to associate with food the odour *o*_2_. Such narrowly related associative learning capacities suggest a common relationship among the underlying association networks. This common relationship is captured by a map between list types, which in the current context is a (graph transformation) map between graph types. We detail this explanation in S3 Text with concrete examples that were inspired by the Rescorla-Wagner model [49] of *classical conditioning* [13]. The examples and theory provided in S3 Text show that the apparent idiosyncratic nature of associative learning in animals is just a narrower form of systematic associative learning that follows naturally from the categorical notion of universal construction. Note that our distinction between wide and narrow forms is primarily for expository purposes: every “wide” construction is a “narrow” construction whose list type map is an identity morphism. These examples also serve to further illustrate associative learning as an anamorphism, and show how our category theory approach makes contact with psychological data, vis-a-vis the Rescorla-Wagner model, thereby elucidating how the principle (of universal construction) espoused by our theory of (second-order) systematicity can be tested. In the specific case of classical conditioning, the universal construction involves a common morphism shared by the anamorphisms capturing the associative learning processes as instances of classical conditioning, which can be tested within the same general approach given in the section “Empirical tests for universal constructions”. Specifically, if one ablates the network component corresponding to the common morphism, then all associative learning capacities purported to factor through that common morphism would no longer be available.

The classical conditioning example also illustrates the general point that there is considerable flexibility within the coalgebraic approach to accommodate different forms of second-order systematicity. Our theory of second-order systematicity need not be committed to a particular model of classical conditioning (see [50] for a review of computational models). Rather, our theory is committed to a particular way in which such associative learning processes are related, i.e., via a common final coalgebra/anamorphism. A final coalgebra captures relatively little common structure—a common (co)recurrent process over a specific internal (e.g., association network) state space—as one may expect, given very little in common between the facts being learned or memorized. An anamorphism (type map) captures additional common structure, where there is greater overlap among the stimuli and responses being associated. On the one hand, this flexibility does not imply that the theory can make arbitrary predictions. The predictions that follow are determined by the scheme already given for initial or terminal objects, since every universal construction can be considered as such. On the other hand, this flexibility raises the question of what principle determines the choice of type map. Evolutionary principles influence species-specific learning biases [46]. A possible corresponding category theory principle is to consider optimization as a universal construction in a category that consists of other categories. These object categories are indexed by the sets of associative networks, just mentioned, and optimization is with respect to some corresponding fitness function (see S3 Text, and also the final subsection).

### Auxiliary versus ad hoc assumption

As we have seen, the scope of a collection of systematically related learning capacities is also determined by list type, i.e. the object *G* in the first diagram (“Network state as external input” section) and the third diagram (“Network state as internal input” section). The flexibility with which *G* is determined seems to suggest that it can be adjusted in an arbitrary, ad hoc manner to fit any collection of associative learning capacities. However, the determination of *G* is not ad hoc, by the criteria for an ad hoc assumption that guides the development of an explanation for (first-order and second-order) systematicity [3].

Ad hoc assumptions are auxiliary assumptions that are: (1) motivated only to fit the data at hand, (2) not verifiable independently of verifying the theory, and (3) unconnected to the theory’s core principles [3]. The representability assumption is not ad hoc on any count. Firstly, the representability of cues such as words, or shapes is independently motivated by facts such as the capacity to recognize words and shapes, regardless of whether such elements take part in word-shape associations. Secondly, we have already shown that the test of representability proceeds independently of the test for association. Thus, each auxiliary assumption can be verified independently of verifying the (universal constructions-based) theory. Thirdly, every universal construction is (by definition) an arrow between an object and a functor. In category theory, one cannot speak of a morphism without speaking about its domain and codomain objects. Thus, each auxiliary assumption is intimately connected to the theory’s core principle of universal construction. Therefore, the representability assumption is not ad hoc by the explanatory standard for systematicity, and science generally [3].

### Prospective remarks

Ultimately, there is a price to be paid for taking a categorical approach. One cannot employ category theory without (tacitly, at least) specifying the category within which the explanation of systematicity is couched. Yet, what determines the ambient category, or the relevant functor to/from which we obtain the universal construction? To put it another way, what determines the categorical context relative to which a construction is (necessarily) universal? We have not yet answered this question in general. To some extent, the choice is determined by the (“shape” of the) domain of interest. For example, a large class of universal constructions, called *limits*, derive from a *shape* category that consists of (typically) a small number of (content irrelevant) objects and morphisms. For instance, in regard to the systematic ability to represent binary relations [10], the natural choice is the shape category with just two objects and no non-identity morphisms. There is no question of choosing a one-object or three-object shape category, because they pertain to (respectively) unary and ternary, but not binary relations. On the other hand, because limits and other universal constructions are defined at the level of abstract categories, the collections of such constructions are parameterized by the specific (concrete) categories that afford them. Not all categories have limits, or other kinds of universal constructions, but there may still be choices among the categories that do for the problem at hand. One possible approach is higher-order category theory [51], where for example objects are categories, morphisms are functors, whence universal morphisms are universal functors to/from other functors.

This issue of context notwithstanding, category theory offers a significant advance over other approaches for a further reason. Universal construction provides necessary and sufficient conditions for having a collection of related cognitive capacities. Science, generally, strives for theories of the natural world that provide necessary and sufficient conditions for the causal relations it seeks to explain. In this sense, category theory is a *theory* of structure: for a universal construction, see S1 Text (Definition 6), the existence criterion (necessity) means that there is at least one such construction; the uniqueness criterion (sufficiency) means that there is at most one such construction (i.e., there can only be one, if it exists), in contradistinction to classical, connectionist and other approaches, which afford various *models* of those structures. These may well be “tough times to be talking systematicity” [5]. However, category theory appears to offer cognitive science the best hope yet of explaining systematicity in that it makes a principled distinction between core (universal) and auxiliary assumptions (other morphisms). This distinction is, after all, what the explanatory criteria for systematicity demand.

## Supporting Information

S1 Text This text provides the basic category theory needed for our model. (PDF)

S2 Text This text provides an algebraic (recursive) formulation of associative learning for comparison with the coalgebraic (corecursion) approach. (PDF)

S3 Text This text provides an illustration of species-specific associative learning (narrow second-order systematicity). (PDF)
