# Supplementary material for: Second-Order Systematicity of Associative Learning: A Paradox for Classical Compositionality and a Coalgebraic Resolution
Source: PLoS One. 2016 Aug 9;11(8):e0160619. doi: 10.1371/journal.pone.0160619 (PMC4978477; doi:10.1371/journal.pone.0160619)
Supplement: S1 Text — (PDF) [file pone.0160619.s001.pdf]

S1 Text

In the interests of brevity and clarity, we only provide definitions and examples directly pertaining to the model, omitting the (albeit, well known) theorems and lemmas that justify statements. Deeper and broader introductions to category theory and categorical treatments of (co)recursion can be found in many textbooks on the topic (e.g., [1, 2]). In the context of systematicity, this paper builds upon our earlier work [3], and particularly in the context of recursive capacities [4], where further technical details are found.

Definition 1 (Category)**.** A *category* **C** consists of:

- a collection of *objects* (A, B, ...);
- a collection of *morphisms* (f, g, ...), written $f : A \to B$ to indicate that A and B are respectively the *domain* and *codomain* of f, including an identity morphism, denoted $1_A : A \to A$, for each object A in **C**; and
- a *composition* operation that sends a pair of *compatible* morphisms $f : A \to B$ and $g : B \to C$, i.e., where the codomain of f equals the domain of g, to their *composite* morphism, denoted $g \circ f : A \to C$,

that together satisfy the axioms of:

– *associativity*: $h \circ (g \circ f) = (h \circ g) \circ f$; and

– *identity*: $f \circ 1_A = f = 1_B \circ f$ for each f in **C**.

Remark. A *subcategory* of a category **C** is a category **B** whose objects, morphisms, and composition operation are taken from **C**, with composition being restricted to the compatible morphisms in **B**. The requirement that a subcategory be a category implies that the identity and composition morphisms in **B** are also in **C**. The concept of subcategory generalizes the concept of subset.

Example 1 (**Set**)**.** The category **Set** has sets for objects, functions between sets for morphisms, and composition is composition of functions.

Definition 2 (Terminal object)**.** In a category **C**, a *terminal object* is an object, denoted 1, such that for every object Z there exists a unique morphism $u : Z \to 1$.

Remark. In **Set**, any singleton set is a terminal object, whose only element is denoted $*$ when its identity is not required. Other categories may have terminal objects with further internal structure, as we shall see for categories of (co)algebras.

Definition 3 (Isomorphism)**.** An *isomorphism* is a morphism $f : A \to B$ such that their exists a morphism $g : B \to A$ satisfying $f \circ g = 1_B$ and $g \circ f = 1_A$. Morphism g is called the *inverse* of f, and denoted f^{-1}.

Remark. In a category $\mathbf{C}$, the collection of morphisms with domain object A and codomain object B is called a *hom-set*, denoted $\mathrm{Hom}_{\mathbf{C}}(A, B)$. As we shall see, hom-sets play an important role in category theory.

Definition 4 (Functor)**.** A *functor* F from a category $\mathbf{C}$ to a category $\mathbf{D}$, written $F : \mathbf{C} \to \mathbf{D}$, maps each object A in $\mathbf{C}$ to the object $F(A)$ in $\mathbf{D}$ and each morphism $f : A \to B$ in $\mathbf{C}$ to the arrow $F(f) : F(A) \to F(B)$ in $\mathbf{D}$ such that the following axioms are satisfied:

- *identity*: $F(1_A) = 1_{F(A)}$ for each object A in $\mathbf{C}$; and
- *compositionality*: $F(g \circ_{\mathbf{C}} f) = F(g) \circ_{\mathbf{D}} F(f)$ for each pair of compatible morphism (f, g).

That is, a functor is a map that preserves category structure.

Remark. An *endofunctor* is a functor $F : \mathbf{C} \to \mathbf{C}$, i.e., the domain and codomain are the same category $\mathbf{C}$. Endofunctors are used to model (co)recursion.

Example 2 (Right product functor)**.** The *right product functor* $\Pi_B : \mathbf{Set} \to \mathbf{Set}$ sends each set X to the *Cartesian product* $X \times B$ and each function $f : X \to Y$ to the *product of functions* $f \times 1_B : X \times B \to Y \times B, (x, b) \mapsto (f(x), b)$, i.e., $f \times 1_B$ maps (x, b) to $(f(x), b)$.

Remark. The Cartesian product of sets A and B is the set, denoted $A \times B$, consisting of all pairwise combinations of the elements of A and B. The product of functions $f : A \to C$ and $g : B \to D$ is the function, denoted $f \times g : A \times B \to C \times D$, that sends each pair (a, b) in $A \times B$ to the pair $((f(a), g(b))$ in $C \times D$.

Example 3 (Right exponential functor)**.** The *right exponential functor* $\Lambda_B : \mathbf{Set} \to \mathbf{Set}$ sends each set X to the function set X^B, which is the set of functions $\{f : B \to X\}$, and each function $g : X \to Y$ to the function $\Lambda(g) : X^B \to Y^B, f \mapsto g \circ f$.

Example 4 (List)**.** List-related constructions built from a set of elements A are obtained from an endofunctor on the category **Set**, i.e., $F_A : X \mapsto 1 + A \times X, f \mapsto 1_1 + 1_A \times f$, where 1 corresponds to the empty list, and $+$ and $\times$ are (respectively) the *disjoint union* and Cartesian product of sets or functions.

Remark. The disjoint union of sets A and B is the set, denoted $A + B$, that consists of all elements from A and B labeled to identify their set of origin. The disjoint union of functions $f : A \to C$ and $g : B \to D$ is the function, denoted $f + g : A + B \to C + D$, that sends each pair $(1, a)$ in $A + B$ to the pair $(1, f(a))$ in $C + D$; likewise, each pair $(2, b)$ to the pair $(2, g(b))$.

Definition 5 (Natural transformation)**.** A *natural transformation* η from a functor $F : \mathbf{C} \to \mathbf{D}$ to a functor $G : \mathbf{C} \to \mathbf{D}$, written $\eta : F \dot{\to} G$, is a family of **D**-morphisms $\{\eta_A : F(A) \to G(A) | A \text{ is an object in } \mathbf{C}\}$ such that for each morphism $f : A \to B$ in **C** we have $G(f) \circ \eta_A = \eta_B \circ F(f)$, i.e., the following diagram is *commutative* (equational):

$$\begin{array}{ccc} F(A) & \xrightarrow{\eta_A} & G(A) \\ {\scriptstyle F(f)}\downarrow & & \downarrow{\scriptstyle G(f)} \\ F(B) & \xrightarrow[\eta_B]{} & G(B) \end{array} \tag{1}$$

Definition 6 (Final morphism)**.** A *final morphism* from a functor $F : \mathbf{C} \to \mathbf{D}$ to an object X in **D** is a pair (A, ϕ) consisting of an object A in **C** and a morphism $\phi : F(A) \to X$ in **D** such that for every object Z in **Z** and every morphism $f : F(Z) \to X$ in **C** there exists a unique morphism $u : Z \to A$ such that $f = \phi \circ F(u)$, as indicated by commutative diagram

$$\begin{array}{cccc} Z & F(Z) & & \\ {\scriptstyle u}\downarrow & {\scriptstyle F(u)}\downarrow & \searrow^{f} & \\ A & F(A) & \xrightarrow[\phi]{} & X \end{array} \tag{2}$$

Remark. The *dual* of final morphism is *initial morphism*, whose definition is obtained by reversing the directions of the morphisms in the definition of final morphism. A universal morphism is either a final morphism or an initial morphism. In general, category theory concepts are dualized by reversing all the arrows in the definition of the original concept.

Remark. Our use of the expression “final morphism” is a shorthand for the standard expression “universal morphism from an object X to a functor F”; dually, “initial morphism” is a shorthand for “universal morphism from a functor F to an object X” (see [1] for the standard forms).

Definition 7 (Adjunction)**.** An *adjunction* from a category $\mathbf{C}$ to a category $\mathbf{D}$ is a triple, written $(F, G, \epsilon) : \mathbf{C} \rightharpoonup \mathbf{D}$, consisting of a functor $F : \mathbf{C} \to \mathbf{D}$, a functor $G : \mathbf{D} \to \mathbf{C}$ and a natural transformation $\epsilon : F \circ G \dot{\to} 1_{\mathbf{D}}$ such that for each object Y in $\mathbf{D}$, the pair $(G(Y), \epsilon_Y)$ is a final morphism from F to Y, as indicated by the following commutative diagram:

$$\begin{array}{ccccc} X & \quad & F(X) & & \\ {\scriptstyle f}\downarrow & & {\scriptstyle F(f)}\downarrow & \searrow^{g} & \\ G(Y) & & F \circ G(Y) & \xrightarrow[\epsilon_Y]{} & Y \end{array} \tag{3}$$

Remark. The functor F is called the *left adjoint* of functor G, and G is called the *right adjoint* of F. The relationship between F and G is called an *adjoint situation*, denoted $F \dashv G$. The morphism ϵ_Y is the component of the natural transformation ϵ at object Y. Definition 7 emphasizes the natural transformation and universal morphism aspects of adjunctions, cf. diagrams 3 and 2. There are a number of different but equivalent definitions of adjunction (see e.g. [1]).

Example 5 (Product-exponential)**.** The right product functor is left adjoint to the right exponential functor, $\Pi_B \dashv \Lambda_B$, see examples 2 and 3, as indicated by commutative diagram

$$\begin{array}{ccccc} A & \quad & A \times B & & \\ {\scriptstyle \tilde{f}}\downarrow & & {\scriptstyle \tilde{f} \times 1_B}\downarrow & \searrow^{f} & \\ C^B & & C^B \times B & \xrightarrow[eval_C]{} & C \end{array} \tag{4}$$

where $\tilde{f}$ is called the *exponential transpose* of f, and $eval_C$ is the evaluation of each function $\tilde{f}_a \in C^B$, parameterized by $a \in A$, at each $b \in B$, i.e., $eval_C(\tilde{f}_a, b) = f(a, b)$.

Remark. An equivalent definition emphasizes the relationship between hom-sets: an adjunction is a bijection (i.e., one-to-one correspondence) between hom-sets $\mathrm{Hom}_{\mathbf{C}}(X, G(Y))$ and $\mathrm{Hom}_{\mathbf{D}}(F(X), Y)$ that is natural (in the natural transformation sense) in variables X and Y, written $\mathrm{Hom}_{\mathbf{D}}(F(X), Y) \cong$

$\mathrm{Hom}_{\mathbf{C}}(X, G(Y))$, as indicated by diagram

$$\begin{array}{ccc} X & \xmapsto{\;F\;} & F(X) \\ {\scriptstyle f}\downarrow & & \downarrow{\scriptstyle g} \\ G(Y) & \underset{G}{\longleftarrow\!\shortmid} & Y \end{array} \tag{5}$$

Hence, one can think of an adjunction as a kind of isomorphism that is local to hom-sets, but not necessarily global to categories. This aspect will be useful when considering adjunctions in the context of corecursion.

Example 6 (Curry-uncurry)**.** The product-exponential adjoint is familiar in functional programming in the form of the *curry-uncurry* operator, named after Haskell Curry, which converts an n-ary function (i.e., a function of n arguments) to a unary function (i.e., a function of one argument). For instance, the curry of addition, written as the binary function $\mathit{add} : \mathbb{N} \times \mathbb{N} \to \mathbb{N}$, is the unary function $\mathit{addN} : \mathbb{N} \to \mathbb{N}^{\mathbb{N}}$, which takes a number x and returns the addx function: e.g., $\mathit{addN} : 1 \mapsto \mathit{add1}$, where $\mathit{add1} : n \mapsto n + 1$. Uncurry is the inverse of curry. The product and exponential operators appear on either side of the bijection $\mathrm{Hom}_{\mathbf{Set}}(\mathbb{N} \times \mathbb{N}, \mathbb{N}) \cong \mathrm{Hom}_{\mathbf{Set}}(\mathbb{N}, \mathbb{N}^{\mathbb{N}})$ obtained from the product-exponential adjoint. Similarly, a state transition function is a binary function $\tau : A \times S \to S$ from inputs, $a \in A$, and states, $s \in S$, to states, or equivalently a unary function $\tau_S : A \times S^S$ from an input to function between states, as given by the bijection $\mathrm{Hom}_{\mathbf{Set}}(A \times S, S) \cong \mathrm{Hom}_{\mathbf{Set}}(A, S^S)$. We make use of this universal construction in our associative learning model.

Definition 8 (F-coalgebra)**.** An *F-coalgebra* on an endofunctor $F : \mathbf{C} \to \mathbf{C}$ is a pair (A, α) consisting of an object A and a morphism $\alpha : A \to F(A)$ in $\mathbf{C}$.

Remark. For comparison, the dual notions of *F-algebra* and related definitions needed for a categorical treatment of recursion are given in S2 Text. These constructions are obtained by reversing the directions of the morphisms in the corresponding coalgebra-related definitions.

Example 7 (Product function over numbers)**.** Suppose we have the *diagonal functor* $\Delta : \mathbf{Set} \to \mathbf{Set}$, which sends each set A to the pair of sets (A, A) and each function $f : A \to B$ to the pair of functions $(f, f) : (A, A) \to (B, B)$. A coalgebra on this functor is the *product function* $\langle \mathsf{I}_1, \mathit{inc} \rangle : \mathbb{N} \to (\mathbb{N}, \mathbb{N}); a \mapsto (1, a + 1)$, where I_1 is the constant function returning 1, and inc is the increment function.

Example 8 (Product function over lists)**.** Suppose we have the *right product functor* $\Pi_A : \mathbf{Set} \to \mathbf{Set}$, which sends each set X to the Cartesian product of sets $A \times X$ and each function $f : X \to Y$ to the product of functions $(1_A, f) : (A, X) \to (A, Y)$. A coalgebra on this functor is the *product function* $\langle \mathit{head}, \mathit{tail} \rangle : L \to A \times L; h \cdot t \mapsto (h, t)$, where *head* returns the first element of each list, and *tail* returns the rest of the list. Here, L is the set of infinite lists whose elements are taken from the set A.

Definition 9 (F-coalgebra homomorphism)**.** An *F-coalgebra homomorphism* from a coalgebra (B, β) to a coalgebra (A, α) is a morphism $h : (B, \beta) \to (A, \alpha)$ such that $F(h) \circ \beta = \alpha \circ h$, as indicated by the following commutative diagram:

$$\begin{array}{ccc} B & \xrightarrow{\beta} & F(B) \\ {\scriptstyle h}\downarrow & & \downarrow{\scriptstyle F(h)} \\ A & \xrightarrow[\alpha]{} & F(A) \end{array} \tag{6}$$

Example 9 (Repeat forever)**.** For the right product functor Π_A given in Example 8, there is an F-coalgebra homomorphism from the coalgebra $\langle \mathsf{I}_a, 1 \rangle : 1 \to A \times 1$ (cf. Example 7) to the coalgebra $\langle \mathit{head}, \mathit{tail} \rangle$ given in Example 8, i.e., $\mathit{repeat} : 1 \to L$, which returns infinite repetitions of a, as indicated by the following commutative diagram:

$$\begin{array}{ccc} 1 & \xrightarrow{\langle \mathsf{I}_a, 1 \rangle} & A \times 1 \\ {\scriptstyle \mathit{repeat}}\downarrow & & \downarrow{\scriptstyle 1_A \times \mathit{repeat}} \\ L & \xrightarrow[\langle \mathit{head}, \mathit{tail} \rangle]{} & A \times L \end{array} \tag{7}$$

where 1 is the identity function on the terminal object 1, i.e., $1 : * \mapsto *$.

Remark. Example 9 highlights the use of coalgebras for unbounded lists. Coalgebras can also be applied to finite lists using conditional functions to test for terminate conditions, which we will introduce shortly.

Definition 10 (Category of F-coalgebras)**.** Suppose we have an endofunctor $F : \mathbf{C} \to \mathbf{C}$. The *category of F-coalgebras*, denoted $\mathbf{CoAlg}(F)$, has F-coalgebras for objects and F-coalgebra homomorphisms for morphisms. Composition is composition of F-coalgebra homomorphisms.

Definition 11 (Final F-coalgebra)**.** Suppose we have a category of F-coalgebras, $\mathbf{CoAlg}(F)$. A *final F-coalgebra* is an F-coalgebra, denoted (A, fin), such that for every F-coalgebra (B, β) in $\mathbf{CoAlg}(F)$ there exists a unique F-coalgebra homomorphism $h : (B, \beta) \to (A, \mathit{fin})$.

Remark. A final F-coalgebra is a terminal (final) object in the category $\mathbf{CoAlg}(F)$.

Example 10 (Infinite lists)**.** The F-coalgebra $(L, \mathit{head}, \langle \mathit{tail} \rangle)$, given in Example 9, is a final coalgebra for infinite list-related coalgebras.

Definition 12 (Anamorphism)**.** An *anamorphism* is an F-coalgebra homomorphism from an F-coalgebra (B, β) to a final F-coalgebra (A, fin), as indicated by the following commutative diagram:

$$\begin{array}{ccc} B & \xrightarrow{\beta} & F(B) \\ {\scriptstyle h}\downarrow & & \downarrow{\scriptstyle F(h)} \\ A & \xrightarrow[\mathit{fin}]{} & F(A) \end{array} \tag{8}$$

Remark. Anamorphism h is denoted $[\![\beta]\!]$, using *lens brackets* [5], since h is completely determined by β. Anamorphism is also called *unfold*.

Remark. Every anamorphism is the unique morphism component pertaining to a universal construction; every final F-coalgebra is a final morphism (universal construction). Compare Diagram 8 and Diagram 2: object A and morphism fin^{-1} from Diagram 8 instantiate (respectively) object A (and X) and morphism ϕ in Diagram 2; object B and morphism h from Diagram 8 instantiate (respectively) object Z and morphism u in Diagram 2; hence, composite morphism $\mathit{fin}^{-1} \circ F(h)$ from Diagram 8 instantiates morphism f in Diagram 2.

Definition 13 (Conditional function)**.** Suppose we have sets A, B and C, and functions $f : A \to B$ and $g : A \to C$. A *conditional function* is a function consisting of a predicate $p? : A \to \{\text{False}, \text{True}\}$ and two alternative functions $f : A \to B$ and $g : A \to C$, written $(p? \to f, g) : A \to B + C$, that is defined as:

$$(p? \to f, g) : a \mapsto \begin{cases} f(a) & \neg p?(a); \\ g(a) & \text{otherwise.} \end{cases}$$

That is, a function that applies alternative f to argument a if $p?(a)$ is false, otherwise alternative g. Recall that $B + C$ is the disjoint union of sets B and C.

Example 11 (List anamorphism)**.** For list-related constructions built from elements in a set A, we have a category of coalgebras on the endofunctor $F_A : X \mapsto 1 + A \times X$. It can be shown that a final coalgebra

for this category consists of conditional function $(\mathit{empty?} \to \mathsf{I}_*, \langle \mathit{head}, \mathit{tail}\rangle) : L \to 1 + A \times L$, where L is the set of lists constructed from elements of a set A, predicate *empty?* tests for empty list, constant function $\mathsf{I}_* : L \to 1$ returns a fixed element $*$, and product function $\langle \mathit{head}, \mathit{tail}\rangle : L \to A \times L$ returns a pair consisting of the head and the tail of the given list, where the head is the first item in the list, and the tail is the rest of the list. Every anamorphism to this final coalgebra is given by commutative diagram

$$\begin{array}{ccc} X & \xrightarrow{(p?\to\mathsf{I}_*,\langle f,g\rangle)} & 1 + A \times X \\ {\scriptstyle [\![p?\to\mathsf{I}_*,\langle f,g\rangle]\!]}\downarrow & & \downarrow{\scriptstyle 1+1_A\times[\![p?\to\mathsf{I}_*,\langle f,g\rangle]\!]} \\ L & \xrightarrow[(\mathit{empty?}\to\mathsf{I}_*,\langle \mathit{head},\mathit{tail}\rangle)]{} & 1 + A \times L \end{array} \tag{9}$$

Since $(\mathit{empty?} \to \mathsf{I}_*, \langle \mathit{head}, \mathit{tail}\rangle)$ is an isomorphism, whose inverse $[\mathit{empty}, \mathit{cons}]$ sends element $*$ to the empty list and pair (h, t) to the list $h \cdot t$, traversing Diagram 9 from X to L clockwise yields the definition:

$$[\![p? \to \mathsf{I}_*, \langle f, g\rangle]\!] : x \mapsto \begin{cases} [\,] & \neg p?(x); \\ f(x) \cdot [\![\cdots]\!](g(x)) & \text{otherwise.} \end{cases}$$

We also write $[\![p? \to \mathsf{I}_*, \langle f, g\rangle]\!]$ as $\mathit{unfold}(p? \to \mathsf{I}_*, \langle f, g\rangle)$.

Remark. Since $(L, (\mathit{empty?} \to \mathsf{I}_*, \langle \mathit{head}, \mathit{tail}\rangle))$ in Example 11 is a final F-coalgebra, it is also a final morphism, hence a universal morphism. Thus, we have shown that second-order systematicity is subsumed by our explanation for first-order systematicity in that they both derive from universal constructions.

References

1. Mac Lane S (1998) Categories for the working mathematician. Graduate Texts in Mathematics. New York, NY: Springer, 2nd edition.

2. Bird R, de Moor O (1997) Algebra of programming. Harlow, England: Prentice Hall.

3. Phillips S, Wilson WH (2010) Categorial compositionality: A category theory explanation for the systematicity of human cognition. PLoS Computational Biology 6: e1000858.

4. Phillips S, Wilson WH (2012) Categorial compositionality III: F-(co)algebras and the systematicity of recursive capacities in human cognition. PLoS ONE 7: e35028.

5. Meijer E, Fokkinga M, Paterson R (1991) Functional programming with bananas, lenses, envelopes and barbed wire, Berlin, Germany: Springer-Verlag, volume 523 of *Lecture Notes in Computer Science*. pp. 125–144.
