# Supplementary material for: Second-Order Systematicity of Associative Learning: A Paradox for Classical Compositionality and a Coalgebraic Resolution
Source: PLoS One. 2016 Aug 9;11(8):e0160619. doi: 10.1371/journal.pone.0160619 (PMC4978477; doi:10.1371/journal.pone.0160619)
Supplement: S2 Text — (PDF) [file pone.0160619.s002.pdf]

S2 Text

Conceptually, the learning of associations can be regarded as a (second-order) function that takes a list of pairs and returns a network (graph) of associations, where each node of the graph represents an associated entity and each (directed) edge is labeled with the strength of association from the source associate to the target associate. Note that the returned graph also represents a (first-order) function, which justifies regarding the learning of associations as a second-order function (second-order cognitive capacity). Lists can be built up inductively—a list is either the empty list, or an item prepended to a list. This inductive construction provides the basis for a recursive model of systematic learning of paired associates, i.e., if there are no more paired associates, then return the constructed network, else add the current paired associate to the network obtained from the learning of the remaining list of associates. The following two examples provide a conceptual guide to this (dual) algebraic approach, and also motivate our coalgebraic model.

Example 1 (Summing)**.** The summing of a list of numbers can be performed by a type of function called a *fold* over lists, denoted $\mathit{fold}(0,+)$, which is an instance of a *catamorphism* [1]. The first argument, 0, assigns the result of zero to the empty list, and the second argument, +, adds the head (first element) of the list to the fold of the tail (remaining elements) of the list. Thus, for example,

$$\begin{aligned}
\mathit{fold}(0,+)[1,2,3] &= 1 + \mathit{fold}(0,+)[2,3] \\
&= 1 + (2 + \mathit{fold}(0,+)[3]) \\
&= 1 + (2 + (3 + \mathit{fold}(0,+)[\,])) \\
&= 1 + (2 + (3 + 0)) = 6.
\end{aligned}$$

Example 2 (Counting)**.** Counting list elements is computed by the catamorphism, $\mathit{fold}(0, \mathit{inc}) : L \to \mathbb{N}$, where the first argument, 0, assigns the result of zero to the empty list, and the second argument, *inc*, ignores the head (first element) of the list and increments the count of the tail (remaining elements) of the list (cf. Diagram 2). In other words, the count of a list is either 0, for the empty list, or one plus the

count of the rest (tail) of the list. For instance,

$$
\begin{aligned}
& \mathit{fold}(0, \mathit{inc})[a, b, c] \\
= \quad & 1 + \mathit{fold}(0, \mathit{inc})[b, c] \\
= \quad & 1 + (1 + \mathit{fold}(0, \mathit{inc})[c] \\
= \quad & 1 + (1 + (1 + \mathit{fold}(0, \mathit{inc})[\,])) \\
= \quad & 1 + (1 + (1 + 0)) \\
= \quad & 3.
\end{aligned}
$$

A categorical treatment of recursion starts with a suitable endofunctor $F : \mathbf{C} \to \mathbf{C}$ from which we construct the category $\mathbf{Alg}(F)$, whose objects are F-algebras, i.e., the pairs (X, ϕ) consisting of objects X and morphisms $\phi : F(X) \to X$ in $\mathbf{C}$, and morphisms are F-algebra homomorphisms, i.e., the morphisms $h : (X, \phi) \to (Y, \psi)$ such that $h \circ \phi = \psi \circ F(h)$. An initial algebra in $\mathbf{Alg}(F)$ is an F-algebra (A, α) such that for each F-algebra (X, ϕ) in $\mathbf{Alg}(F)$ there exists a unique F-algebra homomorphism $u : (A, \alpha) \to (X, \phi)$, called a catamorphism and denoted $(\!|\phi|\!)$, since it is uniquely determined by ϕ [1]. Thus an initial algebra is an *initial object* (dual to terminal object) in $\mathbf{Alg}(F)$.

In greater detail, a catamorphism $k : (A, \mathit{in}) \to (B, \beta)$ from *initial F-algebra* (A, in) to F-algebra (B, β) as indicated by

$$
\begin{array}{ccc}
F(A) & \xrightarrow{\ \mathit{in}\ } & A \\
{\scriptstyle F(k)}\Big\downarrow & & \Big\downarrow{\scriptstyle k} \\
F(B) & \xrightarrow[\ \beta\ ]{} & B
\end{array}
\tag{1}
$$

Catamorphism k is denoted $(\!|\beta|\!)$, by *banana brackets* [2], since k is determined by β. Catamorphism is also called *fold*. For lists built from elements of A, catamorphisms are given by

$$
\begin{array}{ccc}
1 + A \times L & \xrightarrow{\ [\mathit{empty}, \mathit{cons}]\ } & L \\
{\scriptstyle 1 + 1_A \times (\!|\mathsf{I}_v, f|\!)}\Big\downarrow & & \Big\downarrow{\scriptstyle (\!|\mathsf{I}_v, f|\!)} \\
1 + A \times X & \xrightarrow[\ [\mathsf{I}_v, f]\ ]{} & X
\end{array}
\tag{2}
$$

where $\mathit{empty} : * \mapsto [\,]$ returns the empty list, $\mathit{cons} : (h, t) \mapsto h \cdot t$ returns the list with (head) element h prepended to (tail) list t, and I_v assigns the value v to the empty list. The morphism component of

an initial algebra is an isomorphism. For $[\mathit{empty}, \mathit{cons}]$, the inverse is $(\mathit{empty}? \rightarrow \mathsf{I}_*, \langle \mathit{head}, \mathit{tail} \rangle)$. Thus, traversing Diagram 2 from L to X counterclockwise yields the following definition:

$$(\!|\mathsf{I}_v, f|\!) : \begin{cases} [\,] & \mapsto v; \\ h \cdot t & \mapsto f(h, (\!|\mathsf{I}_v, f|\!)(t)). \end{cases}$$

We also write $(\!|\mathsf{I}_v, f|\!)$ as $\mathit{fold}(\mathsf{I}_v, f)$.

For our purposes we employ an endofunctor on the category **Set** for list-related constructions, i.e., $F_A : X \mapsto 1 + A \times X$, where A is the set of possible list elements, 1 is the *terminal object* (associated with the empty list), and operators $\times$ and $+$ are the *Cartesian product* and *disjoint union* of sets. Recall that the Cartesian product $A \times B$ is the set of all pairwise combinations of the elements of A and B. The disjoint union $A + B$ is the set of all elements from either A or B labeled to identify their set of origin. The initial algebra is $(L, [\mathit{empty}, \mathit{cons}])$, where L is the set of lists constructed from the elements of A, *empty* is the function that creates the empty list, and *cons* is the function that prepends an item to a list to create a new list. Every catamorphism $(\!|\mathsf{I}_v, f|\!)$ from the initial algebra in this category has the form given by the following (commutative) diagram:

$$\begin{array}{ccc} 1 + A \times L & \xrightarrow{[\mathit{empty}, \mathit{cons}]} & L \\ {\scriptstyle 1+1_A \times (\!|\mathsf{I}_v, f|\!)}\downarrow & & \downarrow{\scriptstyle (\!|\mathsf{I}_v, f|\!)} \\ 1 + A \times X & \xrightarrow[{[\mathsf{I}_v, f]}]{} & X \end{array} \tag{3}$$

where function I_v assigns the value v to the empty list.

The function $\mathit{fold}(0, +)$ is an instance of the catamorphism given by Diagram 3, i.e., $\mathsf{I}_v = \mathsf{I}_0$, the constant function that returns zero, $f = +$, and hence $(\!|\mathsf{I}_v, f|\!) = \mathit{fold}(0, +)$. Object $A = X = \mathbb{N}$, the set of natural numbers.

For our model of learned associations, we instantiate Diagram 3 as follows:

- A is the set of paired associates $P = \{(a, b) \in S \times S\}$, where a and b are (respectively) the source and target entities from a set of entity representations S;
- X is the set of (labeled) directed graphs (association networks) G, where each graph $g \in G$ is a pair (E, V) consisting of a set of edges E and a set of vertices V, and each edge is a triple (s, σ, t),

where s and t are the source and target vertices and σ is the strength of association;

- I_v is the function I_e that assigns the empty list to the empty graph e; and
- f is the function *assoc* that takes a paired associate and a network graph to returns a network graph.

Hence, our model for learned associations is the catamorphism $(\!|\mathsf{I}_e, \mathit{assoc}|\!) : L \to G$. The function $\mathit{assoc} : P \times G \to G$ creates a network of associations from a paired associate and an association network. Informally, if the graph does not contain an edge corresponding to the given pair, then a new edge with an initial strength of association is added to the graph, else the strength of association for the existing edge is updated.

A simple example serves to illustrate the mechanism of associative learning as a catamorphism. Suppose we have the following list of paired associates: [(bread, butter), (fork, knife), (knife, butter)]. Observe that the catamorphism $(\!|\mathsf{I}_e, \mathit{assoc}|\!) : L \to G$ is obtained from the composition of three functions by traversing Diagram 3 from L to $X = G$ counterclockwise, i.e., (1) $[\mathit{empty}, \mathit{cons}]^{-1}$, (2) $1 + 1_A \times (\!|\mathsf{I}_v, f|\!)$, and (3) $[\mathsf{I}_v, f]$. The first function pulls apart the list of paired associates. This inverse function is guaranteed to exist because $[\mathit{empty}, \mathit{cons}]$ is an isomorphism. The second function, which contains the catamorphism, is also (recursively) obtained by the composition of three functions whenever the list is not empty. The third function constructs the associative network from the current paired associates and the network (recursively) constructed from the previous list. Thus, for our example, the pair (bread, butter) is added to the network constructed from the list [(fork, knife), (knife, butter)], which is constructed by adding (fork, knife) to the network constructed from the list [(knife, butter)], which is constructed by adding (knife, butter) to the network constructed from the empty list, which is just the empty graph. So, the catamorphism constructs the following network, with associative strengths σ_i:

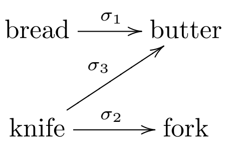

(4)

Second-order systematicity says that if we can learn one collection of word associations (e.g., in English) then we can learn another collection of associations (e.g., in Japanese), assuming of course capacity for English and Japanese words. Such instances of associative learning are catamorphisms from

the same initial algebra, as described by Diagram 3, i.e., replace X, which is instantiated as the set of graphs on English words in the discussion above, with the set of graphs on Japanese words. Moreover, initial algebras are instances of universal morphisms, specifically initial morphisms.

References

1. Bird R, de Moor O (1997) Algebra of programming. Harlow, England: Prentice Hall.

2. Meijer E, Fokkinga M, Paterson R (1991) Functional programming with bananas, lenses, envelopes and barbed wire, Berlin, Germany: Springer-Verlag, volume 523 of *Lecture Notes in Computer Science*. pp. 125–144.
