# Supplementary material for: Second-Order Systematicity of Associative Learning: A Paradox for Classical Compositionality and a Coalgebraic Resolution
Source: PLoS One. 2016 Aug 9;11(8):e0160619. doi: 10.1371/journal.pone.0160619 (PMC4978477; doi:10.1371/journal.pone.0160619)
Supplement: S3 Text — (PDF) [file pone.0160619.s003.pdf]

S3 Text

In this text, we show how our coalgebraic approach is compatible with narrower forms of second-order systematicity. Additional category theory is given in the appendix.

Modality-delimited associative learning

Recall from S1 Text that the universal construction for corecursion in regard to the functor $F_A : X \mapsto 1 + A \times X$, parameterized by the object (set of elements) A, is the final coalgebra that consists of the set of lists L (of type A) together with the morphism that constructs such lists. So, universal morphisms in this context are indexed by the list type A. The universal construction in regard to the functor $F_B : X \mapsto 1 + B \times X$ is a different final coalgebra, i.e., the set of lists of type B together with the morphism for constructing them. In the context of systematicity of associative learning, the type of the list is the set of possible association graphs, G. Hence, a different set of association graphs yields a different final coalgebra. In general, given a family of sets of associative graphs $\mathcal{G} = \{G_i\}$, where i ranges over some index set I, we have a collection of categories, hence final coalgebras (terminal objects in such categories) indexed by i, and for each $i \in I$ we have the anamorphisms indicated by the following commutative diagram (cf. S1 Text, example 11):

$$\begin{array}{ccc}
X & \xrightarrow{(p? \to \mathsf{I}_*, \langle f, g \rangle)} & 1 + G_i \times X \\
{\scriptstyle [\![p? \to \mathsf{I}_*, \langle f, g \rangle]\!]} \downarrow & & \downarrow {\scriptstyle 1 + 1_{G_i} \times [\![p? \to \mathsf{I}_*, \langle f, g \rangle]\!]} \\
L & \xrightarrow[(empty? \to \mathsf{I}_*, \langle head, tail \rangle)]{} & 1 + G_i \times L
\end{array} \tag{1}$$

Hence, the anamorphisms modeling associative learning capacities specialize to the maps indicated in the following commutative diagram:

$$\begin{array}{ccc}
P \times G_i & \xrightarrow{(e? \to \mathsf{I}_*, \langle \mu_i, \nu_i \rangle)} & 1 + G_i \times (P \times G_i) \\
{\scriptstyle [\![e? \to \mathsf{I}_*, \langle \mu_i, \nu_i \rangle]\!]} \downarrow & & \downarrow {\scriptstyle 1 + 1_{G_i} \times [\![e? \to \mathsf{I}_*, \langle \mu_i, \nu_i \rangle]\!]} \\
L & \xrightarrow[(empty? \to \mathsf{I}_*, \langle head, tail \rangle)]{} & 1 + G_i \times L
\end{array} \tag{2}$$

where the merge and next state functions, $\mu_i : P \times G_i \to G_i$ and $\nu_i : P \times G_i \to P \times G_i$, are also indexed since their domains/codomains involve G_i. Other functions involving G_i in both diagrams are

also indexed by i, but we omit relabeling them to reduce clutter.

For the purpose of illustrating narrow, species-specific, second-order systematicity of associative learning, suppose we have a family of associative network sets $\mathcal{G} = \{G_\xi | \xi \in C \times O \times T\}$, where sets C (colour), O (odour), and T (tone) contain the parameters indicating the absence or presence of particular neuronal connections. For simplicity, we suppose that C, O, and T are the same boolean set $\{0, 1\}$, and ξ is interpreted as a sequence of "genetic switches" for turning off/on connections. For example, $\xi = 110$ indicates connections from neurons representing colours to neurons representing foods, connections from neurons representing odours to neurons for foods, but no connections from neurons representing tones to foods. So, the set of association networks G_{110} corresponds to the set of possible neural network connection weights from colour to food neurons, W_{CF}, and odour to food neurons, W_{OF}, but not from tone to food neurons, since there are no such connections. This illustration can be extended to other types of cues and associates.

Given that rats can learn to associate odours with foods, but not colours or tones [1], we have $\xi_{Rat} = 010$, and the set of association networks $G_{\xi_{Rat}}$. The merge function $\mu_{\xi_{Rat}}$ is defined in regard to updating odour-food associations, but not colour-food or tone-food associations. Thus, we have a coalgebra (connection strength update rule) for one set of odour-food associations and a coalgebra for another set of odour-food associations, but no coalgebra for colour-food and tone-food associations (since there are no connections to update), and their systematicity of associative learning derives from the same final coalgebra pertaining to $G_{\xi_{Rat}}$ that is given in diagram 2, as we show in the next section. The same kind of derivation applies to other cases, e.g., quails, which can learn to associate colours to foods, but not odours or tones [1]. In this case, we have $\xi_{Quail} = 100$ and $G_{\xi_{Quail}}$, yielding a different form of narrow, second-order systematicity.

Narrow (within-modality) second-order systematicity

A narrower form of second-order systematicity pertains to stimuli within a particular sensory modality, e.g., having the capacity to learn to associate odour o_1 with food if and only if there is the capacity to learn to associate odour o_2 with food. These forms also derive from a universal construction by a map between list types. In this section, we provide a concrete example inspired by the Rescorla-Wagner model [2] of *classical conditioning* [3], with the purpose of showing how such associative learning processes

are systematically related, though our explanation (in general) does not depend on this model.

Rescorla-Wagner model of classical conditioning

Classical conditioning is a form of associative learning whereby a subject learns to associate a previously neutral stimulus, called the *conditioned stimulus* (CS), by pairing it with a stimulus, called the *unconditioned stimulus* (US), that (innately) elicits a behavioural response. A response elicited by a US is called the *unconditioned response* (UR), and a response elicited by a CS is called a *conditioned response* (CR). For example, food (US) elicits salivation (UR). Repeated pairing of a tone (CS) with food elicits salivation (CR) when stimulated by the tone without the food (classical conditioning). The understanding of the nature of classical conditioning has changed over the years [4], and there does not appear to be a theory that accounts for all the relevant data [5, 6]. However, we use the well-known Rescorla-Wagner model [2] for its simplicity and power in accounting for basic phenomena, despite its shortcomings [7], as a simple concrete example of how our coalgebraic accounts for narrow second-order systematicity of associative learning.

The core of the Rescorla-Wagner model is the equation

$$\Delta V = \alpha\beta(\lambda - V) \tag{3}$$

which says that associative learning proceeds by changing the strength of association, V, between the CS and the US by an amount ΔV that is determined by learning rates α and β for the CS and US, respectively, and the limit of associative strength, λ. Generally, for n conditioned stimuli $\{\mathrm{CS}_i | 1 \leq i \leq n\}$ paired with a US, associative learning is governed by the equation

$$\Delta V_i = \alpha_i \beta(\lambda - \Sigma_{i=1}^{n} V_i). \tag{4}$$

For simplicity and to avoid clashes with symbols used in coalgebra diagrams, we treat the learning rate parameters, α_i and β, as a single parameter ρ that lies within the range 0 to 1, i.e., $0 < \rho = \alpha\beta < 1$. Equation 4 is further simplified by setting $\lambda = 1$ when the US is present, and $\lambda = 0$ when the US is not present, so that the association decays if US is absent. Each strength of association V_i is set to 0 prior to learning.

The Rescorla-Wagner model explains a number of core phenomena, including *acquisition* and *extinction* of associations, and *blocking* of associative learning by a prior association [2]. See [7] for a list of successes, as well as failures of the model.

Acquisition In the presence of a US, the CS receives an increase in associative strength, $\Delta V = (1-\rho)V$. The strength V at time step t is given by the power function $V(t) = 1 - \rho^t$ for V initially 0. Hence, associative strength increases asymptotically to 1.

Extinction In the absence of a US, the CS receives a decrease in associative strength, $\Delta V = -\rho V$, in which case, strength is given by the power function $V(t) = \rho^t$ for V initially 1. Hence, associative strength decreases asymptotically to 0.

Blocking In the presence of an existing (saturated) association between CS1 and the US, i.e. $V_1 = 1$, a (second) CS2 without prior associative learning, i.e., $V_2 = 0$, is blocked from association with the same US by the lack of available associative capacity, i.e., $\Delta V_2 = \rho(1 - V_1 + V_2) = \rho(1 - 1 + 0) = 0$.

Second-order systematicity of classical conditioning

Second-order systematicity of classical conditioning has a familiar form: one has the capacity for classical conditioning of a CS with a US if and only if one has the capacity for classical conditioning of a CS$'$ with the same US. We show that this form of second-order systematicity also has an explanation in terms of universal constructions with regard to coalgebras. First, we translate the Rescorla-Wagner model into a graphical model inspired by the formal equivalence between the Rescorla-Wagner model and the Widrow-Hoff rule for training network weights [8]. Then, we show that the associative learning processes that afford classical conditioning in both cases factor through a common process that constitutes a universal construction (final morphism).

The Rescorla-Wagner model for classical conditioning of the (CS, US) pair translates into the following graphical (network) model:

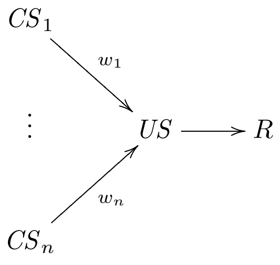

(5)

where each conditioned stimulus CS_i, constituting the CS, is represented by the node CS_i, the unconditioned stimulus US is represented by the node US, the response is represented by the node R, and associative strength V_i is represented by the label (weight) w_i on the corresponding directed edge. The edge corresponding to the association from US to R is unlabeled, since this association is fixed for present purposes. For simplicity, we assume that the absence or presence of a stimulus corresponds to a node activation of 0 or 1, respectively, and that an unconditioned response (UR) and a conditioned response (CR) are just differing response activations r at response node R. For a (CS, US) pair, network weights are updated by the Widrow-Hoff rule, which corresponds to equation 4 of the Rescorla-Wagner model. That is the equation

$$\Delta w_i = \rho(us - \Sigma_{i=1}^{n} w_i cs_i) \tag{6}$$

where us is the activation corresponding to the absence or presence of the US, cs_i is the activation corresponding to the absence or presence of each CS_i constituting the CS, w_i are the weights corresponding to associative strengths, and ρ is the corresponding learning rate parameter.

Next, we provide a coalgebraic account of classical conditioning that incorporates the Rescorla-Wagner model in the network form that we have just given, and show how the associated anamorphisms factor through a common final morphism, thus explaining this instance of second-order systematicity in terms of a universal construction. The anamorphism given in diagram 2 is instantiated as follows.

- A list in P is a list of pairs $ps = (cs, us)$, where $cs = (cs_i)_{i \in \{1,\ldots,n\}}$ is an n-tuple of activations corresponding to the conditioned stimuli constituting the CS, and us is the activation corresponding to the US.
- The set of graphs G_i is the set of stimulus-response networks G_{sr}, where each network $g_{sr} \in G_{sr}$ has the form given by diagram 5 and so is identified with its n-tuple weight state $w = (w_i)_{i \in \{1,\ldots,n\}}$.
- The coalgebra $(e? \to \mathsf{I}_*, \langle \mu_i, \nu_i \rangle)$ is instantiated with predicate function $e?$, merge function μ_{sr}, and the next function ν_{sr} pertaining to G_{sr}. The predicate function (test for the empty list of pairs) and next function (return the next network state) are essentially the same as those given in S1 Text, definition 13). The merge function (weight update) is defined below.
- The final coalgebra $(\mathit{empty}? \to \mathsf{I}_*, \langle \mathit{head}, \mathit{tail} \rangle)$ is essentially the final coalgebra given in S1 Text, example 11.

The merge function $\mu_{sr} : P \times G_{sr} \to G_{sr}$ is defined as

$$\mu_{sr} : (ps, w) \mapsto w + (w_i + c(us - \Sigma w_i cs_i))_{i \in \{1,\ldots,n\}}. \tag{7}$$

The anamorphism just specified is indicated by the following commutative diagram:

$$\begin{array}{ccc} P \times G_{sr} & \xrightarrow{(e? \to \mathsf{!}_*, \langle \mu_{sr}, \nu_{sr} \rangle)} & 1 + G_{sr} \times (P \times G_{sr}) \\ {\scriptstyle [\![e? \to \mathsf{!}_*, \langle \mu_{sr}, \nu_{sr} \rangle]\!]} \downarrow & & \downarrow {\scriptstyle 1 + 1_{G_{sr}} \times [\![e? \to \mathsf{!}_*, \langle \mu_{sr}, \nu_{sr} \rangle]\!]} \\ L & \xrightarrow[(empty? \to \mathsf{!}_*, \langle head, tail \rangle)]{} & 1 + G_{sr} \times L \end{array} \tag{8}$$

Since the anamorphic model employs the same weight update rule as the Rescorla-Wagner model, the anamorphic model inherits the same accounts of basic phenomena, including acquisition, extinction, and blocking, as well as the same shortcomings of the Rescorla-Wagner model [7].

We now show that each anamorphism indicated by diagram 8 is uniquely composed of another anamorphism, which is a final morphism for this universal construction. For the (CS, US) pair, a graph $g_{sr} \in G_{sr}$ is composed of a CS-US subgraph $g_s \in G_s$ and the common US-R subgraph $g_r \in G_{sr}$. The subgraph g_s consists of the unconditioned and conditioned stimulus nodes (CS_i and US) and the stimulus-stimulus connections, labeled as w_i. The subgraph g_r consists of just the US and R nodes and the single (unlabeled) edge. Likewise, for the (CS$'$, US) pair, we have the graph $g'_{sr} \in G_{sr}$, which is composed of a CS$'$-US subgraph $g'_s \in G_s$ and the same US-R subgraph g_r. Since g_r is constant across networks, we define a graph transformation map $g : G_s \to G$ that adjoins to each subgraph $g_s \in G_s$ the subgraph g_r at the common node, US, to form the graph $g_{sr} \in G_{sr}$. Thus, g is a (graph) type map, hence $g^* : L_{G_s} \to L_{G_{sr}}$ is a list map: a map from lists of graph type G_s to lists of graph type G_{sr} (see Appendix, definition 3). By theorem 4, we have (L_{G_s}, g^*) as a final morphism: every anamorphic version of the Rescorla-Wagner model factors through g^*. The universal construction given in diagram 13 is instantiated as follows.

- The coalgebra (X, α) is the coalgebra for the updating the stimulus-stimulus graph $(P \times G_s, \alpha_s)$, where $\alpha_s = (e? \to \mathsf{!}_*, \langle \mu_s, \nu_s \rangle)$. Merge and next functions, μ_s and ν_s, specialize to graph type G_s.
- The final coalgebra (L_A, fin_A) is specialized to the final coalgebra $(L_{G_s}, \mathit{fin}_{G_s})$. Likewise, the final coalgebra (L_B, fin_B) is specialized to the final coalgebra $(L_{G_{sr}}, \mathit{fin}_{G_{sr}})$.
- The type change function $f : A \to B$ is the graph type change function $g : G_s \to G_{sr}$. Hence, the

function $\hat{f}_X$ is the function $\hat{g}_{G_s}$, and the list map morphism f^* is the list map morphism g^*.

The universal construction just specified is indicated by the following commutative diagram:

$$\begin{array}{ccccc} (P \times G_s, \alpha_s) & \quad & (P \times G_s, \hat{g}_{G_s} \circ \alpha_s) & \xrightarrow{\llbracket \hat{g}_{G_s} \circ \alpha_s \rrbracket} & (L_{G_{sr}}, \mathit{fin}_{G_{sr}}) \\ \downarrow{\scriptstyle \llbracket \alpha_s \rrbracket} & & \downarrow{\scriptstyle \mathcal{F}(\hat{g}_{G_s}) \llbracket \alpha_s \rrbracket} & \nearrow{\scriptstyle g^*} & \\ (L_{G_s}, \mathit{fin}_{G_s}) & & (G_s, \hat{g}_{G_s} \circ \mathit{fin}_{G_s}) & & \end{array} \tag{9}$$

For classical conditioning of (CS, US), the list of pairs P is the list of pairs P_{CS}: the sequence of conditional-unconditional (CS, US) stimulus pairings. From diagram 9, the corresponding anamorphism that models this instance of classical conditioning is $\llbracket \hat{g}_{G_s} \circ \alpha_s \rrbracket_{CS} = g^* \circ \mathcal{F}(\hat{g}_{G_s}) \llbracket \alpha_s \rrbracket_{CS}$. For classical conditioning of (CS$'$, US), the list P is $P_{CS'}$: the sequence of conditional-unconditional (CS$'$, US) stimulus pairings. The corresponding anamorphism is $\llbracket \hat{g}_{G_s} \circ \alpha_s \rrbracket_{CS'} = g^* \circ \mathcal{F}(\hat{g}_{G_s}) \llbracket \alpha_s \rrbracket_{CS'}$. Both anamorphisms factor through g^*. Hence, this universal construction accounts for this instance of narrow second-order systematicity of associative learning.

Examples

The following examples serve three purposes: (1) to provide a concrete illustration of an anamorphism in the context of associative learning, (2) to show how the Rescorla-Wagner model is compatible with the anamorphic approach, and (3) to illustrate second-order systematicity with regard to classical conditioning. We assume classical conditioning for two previously neutral stimuli (odours), CS1 and CS2, by pairing with a food-related stimulus as the US eliciting some food-related response R (e.g., salivation). The network model shown in diagram 5 is instantiated to $n = 2$, so a network is identified by a pair of weight states (w_1, w_2). We use a learning rate $\rho = 0.5$. In this example, a list of pairings of conditioned and unconditioned stimuli is a list of triples, (CS1, CS2, US), where $-/+$ indicates absence/presence of the stimulus, and 0/1 is the corresponding node activation. For instance, the triple $(+, -, +)$ indicates pairing of CS1, but not CS2 with the US, and the corresponding node activation is $(1, 0, 1)$. So, five repetitions of such a pairing during training is specified by the list $[(+, -, +), (+, -, +), (+, -, +), (+, -, +), (+, -, +)]$. The corresponding anamorphism in diagram 8, i.e. $\llbracket e? \rightarrow \mathsf{l}_*, \langle \mu_{sr}, \nu_{sr} \rangle \rrbracket$, is instantiated to P containing the list $[(1, 0, 1), (1, 0, 1), (1, 0, 1), (1, 0, 1), (1, 0, 1)]$ and the set of graphs $G_{sr} = \{g_w | (w \in W \times W\}$, where the set of possible weights states $W = \{w \in \mathbb{R} | 0 \leq w \leq 1\}$. By traversing diagram 8 counterclockwise,

with the merge function μ_{sr} given as equation 7, we have the mapping $(p, g_{w_0}) \mapsto g_{w_1} \cdot g_{w_2} \cdot g_{w_3} \cdot g_{w_4} \cdot []$, where w_t is given in the corresponding row of table 1. The unfolding of weights for an example of extinction and blocking is also given in table 1.

Phenomenon	Step (t)	(CS1, CS2, US)	Δw_t	w_t
Acqusition	0	$(-,-,-)$	$(0.000, 0)$	$(0.000, 0)$
	1	$(+,-,+)$	$(0.500, 0)$	$(0.500, 0)$
	2	$(+,-,+)$	$(0.250, 0)$	$(0.750, 0)$
	3	$(+,-,+)$	$(0.125, 0)$	$(0.875, 0)$
	4	$(+,-,+)$	$(0.063, 0)$	$(0.938, 0)$
Exinction	5	$(+,-,-)$	$(-0.469, 0)$	$(0.469, 0)$
	6	$(+,-,-)$	$(-0.235, 0)$	$(0.234, 0)$
	7	$(+,-,-)$	$(-0.117, 0)$	$(0.117, 0)$
	8	$(+,-,-)$	$(-0.059, 0)$	$(0.058, 0)$
	9	$(+,-,-)$	$(-0.029, 0)$	$(0.029, 0)$
Blocking	0	$(-,-,-)$	$(0.000, 0)$	$(0.000, 0)$
	1	$(+,-,+)$	$(0.500, 0)$	$(0.500, 0)$
	2	$(+,-,+)$	$(0.250, 0)$	$(0.750, 0)$
	3	$(+,-,+)$	$(0.125, 0)$	$(0.875, 0)$
	4	$(+,-,+)$	$(0.063, 0)$	$(0.938, 0)$
	5	$(+,+,+)$	$(0.031, 0.031)$	$(0.969, 0.031)$
	6	$(+,+,+)$	$(0.000, 0.000)$	$(0.969, 0.031)$

Table 1. Acquisition and extinction of CS1-US association; blocking of CS2-US association.

An example of second-order systematicity is to swap the roles of CS1 and CS2. That is, if one has the capacity for acquisition (likewise, extinction and blocking) with regard to CS1, then one also has the same capacity for acquisition (likewise, extinction and blocking) with regard to CS2, since both capacities are related by the same US-R link, which is captured by the corresponding instantiation of diagram 9. The important and testable prediction is that if the US-R link is ablated, then both learning capacities would disappear. Alternatively, if each capacity was realized by a distinct link to R, then the ablation of one link would leave the capacity corresponding to the other link preserved.

Interchanging CS1 and CS2 is an instance of a routine (counterbalancing) procedure employed to check that observed effects are not due to idiosyncratic arrangements of stimuli. Indeed, psychological experiments tacitly provide support for this kind of narrow systematicity when reporting no significant effects across counterbalanced conditions.

Our universal constructions explanation for narrow second-order systematicity is not committed to the Rescorla-Wagner model. Although the Rescorla-Wagner model captures many of the empirical findings

in regard to classical conditioning, there are also many other pertinent findings that the model does not capture [7]. In particular, recovery from blocking in the absence of further training seems to contradict the monotonic positive relationship between US and R (see [7] for analysis and discussion). Such contradictory findings suggest that a more complex network is required to model the various causal relations between stimulus and response types. However, our methodology, illustrated above for the Rescorla-Wagner model, would be the same, whereby the common network component is captured by a corresponding universal morphism (graph transformation). Though there is considerable flexibility in choice of universal morphism the predictions follow the same scheme for initial/terminal objects, as detailed in the main text (see subsection, "Empirical tests for universal constructions"), as every universal construction can be construed this way.

Modality-specific performance

Another form of species-specific learning bias pertains to associative learning performance. For example, bees learn odour-flower associations more rapidly than colour-flower associations, which are learned more rapidly than shape-flower associations [1]. Evolutionary forces could have configured genes modulating learning rates on the basis of cue reliability: flowers generally maintain a constant odour, whereas colour changes with ambient lighting, and shape depends on viewpoint [1]. A model of second-order systematicity that incorporates this kind of learning bias could be developed by including additional parameters such as the rate of change of associative strength, in the spirit of more sophisticated models of associative learning, as reviewed in [8] for example. However, this kind of learning bias pertains to performance not competence, i.e., bees have the capacity to learn odour-flower associations if and only if they have the capacity to learn colour-flower associations, but performance differs (see Discussion). Similar considerations also apply to differences in learning rates of specific odours [1].

References

1. Gould JL, Marler P (1991) Learning by instinct. In: Wang WSY, editor, The emergence of language: Development and evolution, New York, NY: Freeman, chapter 12. pp. 190–207.

2. Rescorla RA, Wagner AR (1972) A theory of pavlovan conditioning: Variations in the effectiveness of reinforcement and nonreinforcement. In: Black AH, Prokasy WF, editors, Classical Conditioning II: Current Theory and Research, New York, NY: Appleton-Century-Crofts. pp. 64–99.

3. Shettleworth SJ (2010) Cognition, evolution, and behavior. Oxford, UK: Oxford University Press, 2nd edition.

4. Rescorla RA (1988) Pavlovian conditioning—it's not what you think. American Psychologist 43: 151–160.

5. Brandon SE, Vogel EH, Wagner AR (2002) Computational theories of classical conditioning. In: Moore JW, editor, A neuroscientist's guide to classical conditioning, New York, NY: Springer Science+Business Media, LLC, chapter 7. pp. 232–310.

6. Chance P (2008) Learning and behavior: active learning edition. Belmont, CA: Wadsworth, 6th edition.

7. Miller RR, Barnet RC, Grahame NJ (1995) Assessment of the rescorla-wagner model. Psychological Bulletin 117: 363–386.

8. Sutton RS, Barto AG (1996) Toward a modern theory of adaptive networks: expectation and prediction. Psychological Review 88: 135–170.

9. Bird R, de Moor O (1997) Algebra of programming. Harlow, England: Prentice Hall.

10. Meijer E, Fokkinga M, Paterson R (1991) Functional programming with bananas, lenses, envelopes and barbed wire, Berlin, Germany: Springer-Verlag, volume 523 of *Lecture Notes in Computer Science*. pp. 125–144.

11. Awodey S (2010) Category theory. Oxford Logic Guides. New York, NY: Oxford University Press, 2nd edition.

12. Hinze R (2011) Type fusion. In: Johnson M, Pavlovic D, editors, Algebraic Methodology and Software Technology, Berlin, Germany: Springer, volume 6486 of *Lecture Notes in Computer Science*. pp. 92–110.

Appendix: Typed list coalgebra

In this appendix, we provide basic category theory definitions and theorems pertaining to typed list coalgebras, as needed for our coalgebraic account of narrow second-order systematicity (see also [9, 10]).

Definition 1 (Cartesian product of categories)**.** The *Cartesian product of categories* $\mathbf{A}$ and $\mathbf{B}$ is the category, written $\mathbf{A} \times \mathbf{B}$, that has for objects pairs of objects (A, B) for each object A in $\mathbf{A}$ and B in $\mathbf{B}$, and for morphisms pairs of morphisms $(f, g) : (A, B) \to (C, D)$ for each morphism $f : A \to C$ in $\mathbf{A}$ and $g : B \to D$ in $\mathbf{B}$. Composition is element-wise.

Remark. Cartesian product of categories and *bifunctor*, defined next, generalize Cartesian product of sets and bivariate function (see [11] for an introduction).

Definition 2 (Bifunctor)**.** A *bifunctor* is a functor from the Cartesian product of a pair of categories $\mathbf{A}$ and $\mathbf{B}$ to a category $\mathbf{C}$, written $F(-, -) : \mathbf{A} \times \mathbf{B} \to \mathbf{C}$.

Remark. Setting the first argument of $F(-, -) : \mathbf{A} \times \mathbf{B} \to \mathbf{C}$ to be the object $A \in \mathbf{A}$ yields the functor $F(A, -) : \mathbf{B} \to \mathbf{C}$. Setting the second argument of $F(-, -)$ to be the object $B \in \mathbf{B}$ yields the functor $F(-, B) : \mathbf{A} \to \mathbf{C}$.

Example 1 (Typed coalgebra)**.** Suppose we have a bifunctor $F(-, -) : \mathbf{Set} \times \mathbf{Set} \to \mathbf{Set}; (A, X) \mapsto 1 + A \times X$, and a map between sets (considered as types) $f : A \to B$. Then, we have:

- an endofunctor $F(A, -)$ and corresponding final coalgebra (L_A, fin_A), and
- a morphism $F(-, X)(f) = 1 + f \times 1_X : 1 + A \times X \to 1 + B \times X$, which we denote $\hat{f}_X$.

Definition 3 (Functor *fmap*)**.** Suppose we have a (type) map $f : A \to B$ and sets of lists L_A and L_B of types A and B, respectively. The functor *fmap* : $\mathbf{Set} \to \mathbf{Set}$ sends each type A to the set of lists of type A, i.e., to L_A, and each type map f to the corresponding map over lists, i.e., $\mathit{fmap} : (f : A \to B) \mapsto (\mathit{fmap}(f) : L_A \to L_B)$. The morphism $\mathit{fmap}(f)$ is also denoted f^*.

Remark. The name *fmap* for this functor, like the names *fold* and *unfold* for catamorphism and anamorphism, is used in the context of a categorical approach to functional programming (see [12]). Notation f^* is derived from the notation used for strings: $a*$ indicates zero of more occurrences of the character a; by analogy, f^* indicates zero or more (element-wise) applications of f to a list, i.e. a list having zero or more elements.

Proposition 1 (Map anamorphism)**.** A list map $f^* : L_A \to L_B$ is an anamorphism.

Proof. The proof follows from the commutative diagram

$$\begin{array}{ccccc} L_A & \xrightarrow{fin_A} & 1 + A \times L_A & \xrightarrow{\hat{f}_{L_A} = 1 + f \times 1_{L_A}} & 1 + B \times L_A \\ \Big\downarrow{\scriptstyle f^* = \llbracket \hat{f}_{L_A} \circ fin_A \rrbracket} & & & & \Big\downarrow{\scriptstyle 1 + 1_B \times \llbracket \hat{f}_{L_A} \circ fin_A \rrbracket} \\ L_B & & \xrightarrow[fin_B]{\hspace{6em}} & & 1 + B \times L_B \end{array} \tag{10}$$

which indicates that list map $f^* = \llbracket \hat{f}_{L_A} \circ fin_A \rrbracket : (L_A, \hat{f}_{L_A} \circ fin_A) \to (L_B, fin_B)$. □

Remark. The dual result, which pertains to catamorphisms, is given in [10], equation 33 (and the next equation corresponds to proposition 1).

Fusion laws

A well-known theorem for anamorphisms, called the *fusion law*, says that the composition of a morphism (coalgebra homomorphism) and an anamorphism is an anamorphism [10].

Theorem 2 (Ana-fusion)**.** Suppose we have an endofunctor $F : \mathbf{C} \to \mathbf{C}$ and the category of coalgebras $\mathbf{CoAlg}(F)$. If $h : (C, \gamma) \to (B, \beta)$ is a coalgebra homomorphism and $\llbracket \beta \rrbracket : (B, \beta) \to (A, fin)$ is an anamorphism in $\mathbf{CoAlg}(F)$, then $\llbracket \beta \rrbracket \circ h = \llbracket \gamma \rrbracket : (C, \gamma) \to (A, fin)$ is an anamorphism in $\mathbf{CoAlg}(F)$, as indicated by the following commutative diagram:

$$\begin{array}{ccccc} & C & \xrightarrow{\gamma} & F(C) & \\ & \Big\downarrow{\scriptstyle h} & & \Big\downarrow{\scriptstyle F(h)} & \\ \llbracket \gamma \rrbracket \ (C \to A) & B & \xrightarrow{\beta} & F(B) & F\llbracket \gamma \rrbracket \ (F(C) \to F(A)) \\ & \Big\downarrow{\scriptstyle \llbracket \beta \rrbracket} & & \Big\downarrow{\scriptstyle F\llbracket \beta \rrbracket} & \\ & A & \xrightarrow[fin]{} & F(A) & \end{array} \tag{11}$$

Proof. The proof follows from the commutativity of diagram 11: the top and bottom squares commute by assumption, and the outer square (rectangle) commutes by the "pasting" of commutative squares. Hence, $\llbracket \beta \rrbracket \circ h$ is a coalgebra homomorphism. The uniqueness of $\llbracket \beta \rrbracket \circ h$ follows from the fact that (A, fin) is the final coalgebra (terminal object). Therefore, $\llbracket \beta \rrbracket \circ h$ is the anamorphism $\llbracket \gamma \rrbracket$. □

Remark. There is an analogous law for catamorphisms, *cata-fusion*: the composition of a catamorphism $(\!|beta|\!)$ and an algebra homomorphism k is a catamorphism $(\!|\gamma|\!)$, i.e., $k \circ (\!|beta|\!) = (\!|\gamma|\!)$, and other kinds of universal constructions [9, 10].

Remark. Since a map over lists is an anamorphism (proposition 1), composition of a coalgebra homomorphism with a map over lists is an anamorphism, as given by the following *Promotion* lemma [10], equation 35.

Lemma 3 (Promotion)**.** Suppose we have the following data:

- a category $\mathbf{C}$,
- endofunctors $F_A : X \mapsto 1 + A \times X$ and $F_B : X \mapsto 1 + B \times X$ on $\mathbf{C}$, hence
- categories of coalgebras $\mathbf{CoAlg}(F_A)$ and $\mathbf{CoAlg}(F_B)$, and their (respective)
- final coalgebras (L_A, fin_A) and (L_B, fin_B),
- a coalgebra (X, α) in $\mathbf{CoAlg}(F_A)$, i.e., $\alpha = p? \to \langle \phi, \psi \rangle$, hence
- an anamorphism $[\![\alpha]\!] : (X, \alpha) \to (L_A, \mathit{fin}_A)$ in $\mathbf{CoAlg}(F_A)$, and
- a (type) map $f : A \to B$ in $\mathbf{C}$.

Every anamorphism $[\![\hat{f}_X \circ \alpha]\!]$ in $\mathbf{CoAlg}(F_B)$ is uniquely composed of a coalgebra homomorphism $[\![\alpha]\!] : (X, \hat{f}_X \circ \alpha) \to (L_A, \hat{f}_{L_A} \circ \mathit{fin}_A)$ and the list map (anamorphism) $f^* = [\![\hat{f}_{L_A} \circ \mathit{fin}_A]\!] : (L_A, \hat{f}_{L_A} \circ \mathit{fin}_A) \to (L_B, \mathit{fin}_B)$, as indicated by the following commutative diagram:

$$
\begin{array}{ccccc}
X & \xrightarrow{\alpha} & 1 + A \times X & \xrightarrow{\hat{f}_X} & 1 + B \times X \\
{\scriptstyle [\![\alpha]\!]}\downarrow & & \downarrow{\scriptstyle 1+1_A \times [\![\alpha]\!]} & & \downarrow{\scriptstyle 1+1_B \times [\![\alpha]\!]} \\
L_A & \xrightarrow{\mathit{fin}_A} & 1 + A \times L_A & \xrightarrow{\hat{f}_{L_A}} & 1 + B \times L_A \\
{\scriptstyle [\![\hat{f}_{L_A} \circ \mathit{fin}_A]\!]}\downarrow & & & & \downarrow{\scriptstyle 1+1_B \times [\![\hat{f}_{L_A} \circ \mathit{fin}_A]\!]} \\
L_B & & \xrightarrow[\mathit{fin}_B]{} & & 1 + B \times L_B
\end{array}
\tag{12}
$$

Proof. The proof follows from the commutativity and uniqueness properties of diagram 12. The uniqueness of coalgebra homomorphism $[\![\alpha]\!]$ and the uniqueness property of anamorphisms implies that $[\![\hat{f}_X \circ \alpha]\!] = [\![\hat{f}_{L_A} \circ \mathit{fin}_A]\!] \circ [\![\alpha]\!]$. □

Remark. We use this lemma to show that a map over lists, f^*, is a universal morphism, i.e., there is a universal way for transforming a list of type A into a list of type B.

Theorem 4 (Type universal construction)**.** Suppose we have endofunctors $F_A : X \mapsto 1 + A \times X$ and $F_B : X \mapsto 1 + B \times X$ on $\mathbf{C}$, hence categories of coalgebras $\mathbf{CoAlg}(F_A)$ and $\mathbf{CoAlg}(F_B)$, and a functor $\mathcal{F}(\hat{f}_X) : \mathbf{CoAlg}(F_A) \to \mathbf{CoAlg}(F_B)$ that sends each coalgebra (X, α) in $\mathbf{CoAlg}(F_A)$ to the coalgebra $(X, \hat{f}_X \circ \alpha)$ in $\mathbf{CoAlg}(F_B)$, and each coalgebra homomorphism $[\![\alpha]\!]$ in $\mathbf{CoAlg}(F_A)$ to the coalgebra homomorphism $[\![\hat{f}_X \circ \alpha]\!]$ in $\mathbf{CoAlg}(F_B)$. There is a final morphism $((L_B, \mathit{fin}_B), f^*)$ from the functor $\mathcal{F}(\hat{f}_X)$ to the object (L_B, fin_B), as indicated in the following diagram:

$$
\begin{array}{ccccc}
(X, \alpha) & \qquad & (X, \hat{f}_X \circ \alpha) & \xrightarrow{\;[\![\hat{f}_X \circ \alpha]\!]\;} & \\
\Big\downarrow{\scriptstyle [\![\alpha]\!]} & & \Big\downarrow{\scriptstyle \mathcal{F}(\hat{f}_X)[\![\alpha]\!]} & & \searrow \\
(L_A, \mathit{fin}_A) & & (L_A, \hat{f}_X \circ \mathit{fin}_A) & \xrightarrow[f^*]{} & (L_B, \mathit{fin}_B)
\end{array}
\tag{13}
$$

Proof. The proof follows from lemma 3, and the functoriality of $\mathcal{F}(\hat{f}_X)$. □

Remark. The universal construction given in diagram 13 says that there is a universal way of changing the type of the lists constructed by anamorphisms, via the type change map $f : A \to B$. In the case of association graphs, A is the set of association graphs, and B could be another type of association graph, in which case f is a graph transformation map. For a more general development of such universal constructions with regard to types, called *type fusion*, see [12].
